# SLC22A8: An indicator for tumor immune microenvironment and prognosis of ccRCC from a comprehensive analysis of bioinformatics

**DOI:** 10.1097/MD.0000000000030270

**Published:** 2022-09-16

**Authors:** Ke Xu, Yuni Wu, Hao Chi, Yunyue Li, Yuchen She, Xisheng Yin, Xin Liu, Bingsheng He, Xiaosong Li, Hongjuan Du

**Affiliations:** a Department of Oncology, Chongqing General Hospital, Chongqing, China; b Clinical Medical College, Southwest Medical University, Luzhou, Sichuan, China; c Queen Mary College, Medical School of Nanchang University, Nanchang, Jiangxi, China; d Department of Oncology, The Affiliated Hospital of Southwest Medical University, Luzhou, Sichuan, China; e Clinical Molecular Medicine Testing Center, The First Affiliated Hospital of Chongqing Medical University, Chongqing, China.

**Keywords:** ccRCC, DNA methylation, immune infiltration, prognostic biomarker, SLC22A8

## Abstract

Clear cell renal cell carcinoma (ccRCC) is one of the most common renal malignancies worldwide. SLC22A8 plays a key role in renal excretion of organic anions. However, its role in ccRCC remains unclear; therefore, this study aimed to elucidate the relationship between SLC22A8 and ccRCC. The The Cancer Genome Atlas-kidney renal clear cell carcinoma cohort was included in this study. The Wilcoxon signed-rank test and logistic regression were used to analyze the relationship between SLC22A8 expression and clinicopathological characteristics. Multifactorial analysis and Kaplan–Meier survival curves were adopted for correlation between SLC22A8 expression and clinicopathological parameters and overall survival. Utilizing the UALCAN database, the correlation of the expression levels of SLC22A8 DNA methylation in ccRCC was explored. Immunological characterization of SLC22A8 regarding the ccRCC tumor microenvironment was carried out by the single sample Gene Set Enrichment Analysis algorithm and the CIBERSORT algorithm. With the CellMiner database, the analysis of the association between SLC22A8 gene expression and drug sensitivity was further performed. Eventually, gene ontology and Kyoto Encyclopedia of Gene and Genome enrichment analyses were applied to identify the functional and signaling pathways involved in SLC22A8. SLC22A8 expression is associated with age, grade, stage, and tumor status. SLC22A8 protein expression levels, phosphorylated protein levels, and DNA methylation expression levels were lower in ccRCC tissues than in normal tissues, and low methylation levels predicted poor overall survival. Comprehensive analysis of tumor immune infiltration and the tumor microenvironment indicated a higher level of overall immunity in the SLC22A8 low expression group. Gene Enrichment Analysis results showed that low expression of SLC22A8 was associated with immune pathways, such as phagocytosis recognition and humoral immune response. SLC22A8 expression was significantly correlated with survival and immune infiltration in ccRCC and can be used as a prognostic biomarker for ccRCC.

## 1. Introduction

According to statistics, by 2020, there will be 19.3 million new cancer cases worldwide, of which kidney cancer will account for 2.2%.^[1]^ Renal cell carcinoma (RCC) is the most common solid tumor in adult kidney cancer; 20% of patients are found to have metastatic RCC at initial diagnosis; 20% of patients with primary nonmetastatic renal cell carcinoma recur after treatment; therefore, the current status of its diagnosis and treatment is not optimistic.^[2]^ RCC includes several pathological types, the most common being ccRCC, which accounts for 70% of all cases.^[3,4]^ In recent years, there has been increasing evidence that different pathological subtypes of RCC are histologically and molecularly heterogeneous and have different prognoses.^[5]^ Therefore, it is particularly important to understand the molecular mechanisms involved, identify biomarkers to support treatment, and determine the prognosis.

The SLC solute transporter family is an essential substance transport system in humans. In the human genealogy, 52 genes are expressed in the SLC gene family, covering >395 genes.^[6]^ SLC22 transporter proteins are among the most studied SLC families. There are several subgroups of the SLC22 family, including the organic anion transporter (OAT), which is responsible for regulating the levels of signaling molecules and metabolites in tissues and body fluids, plays a key role in small-molecule communication between organisms, and is involved in the regulation of local and systemic homeostasis.^[7,8]^ SLC22A8, also known as an OAT protein 3 (OAT3), belongs to the OAT subgroup, is highly expressed in the basolateral membrane of human and rodent proximal renal tubules, and mediates the secretion of exogenous and endogenous anions.^[9]^ OAT3 has been reported to be involved in the transport of several uremic toxins and solutes from the gut microbiota (e.g., CMPF, phenyl sulfate, indole-3-acetic acid), mediates the clearance of several drugs in vivo (e.g., enalapril and β-lactam antibiotics), and is a key molecule affecting drug efficacy and toxicity.^[10–12]^ In recent years, the role of SLC22A8 in tumors has received increasing attention. SLC22A8 is expressed only in HepG2 cells compared to normal liver cells.^[13]^ In contrast, increased protein expression of SLC22A8 in acute lymphoblastic leukemia cells and its mediated clearance of furosemide may in turn lead to an increased risk of clinical treatment failure and drug resistance.^[14]^ However, few studies have been conducted to elucidate how SLC22A8 is involved in the biological process of renal clear cell carcinoma. For this reason, insight into the mechanisms of differential SLC22A8 expression in renal clear cell carcinoma could provide a theoretical basis for the development of new cancer treatment strategies.

DNA methylation, the addition of methyl to the 5′-carbon end of cytosine residues in CpG dinucleotides, is the most common form of epigenetic modification that regulates gene expression and is involved in a variety of biological behaviors of tumors.^[15]^ Studies have shown that altered DNA methylation can regulate the function of oncogenes/antioncogenes, affect the progression of RCC, and classify RCC into different prognostic subtypes based on their differential expression levels.^[16]^ However, the specific mechanisms underlying epigenetic modifications in renal clear cell carcinoma have not been fully elucidated. The tumor microenvironment (TME) contains a large number of extracellular matrix and immune infiltrating cells such as TAMs and neutrophils.^[17]^ The Wnt pathway inhibitor CGX-1321 significantly reduced the tumor load and increased CD8 + T-cell levels in the TME of ovarian cancer.^[18]^ Bufalin inhibits tumor microenvironment-mediated angiogenesis by inhibiting the STAT3 signaling pathway in vascular endothelial cells.^[19]^ Notably, ccRCC has a highly immune infiltrative profile,^[20]^ but the association between TME and the development of renal clear cell carcinoma still needs to be further explored. Therefore, understanding the regulatory mechanisms of molecules in TME is of great value and significance for the diagnosis, prognosis, and treatment of ccRCC.

To the best of our knowledge, no study has examined SLC22A8 methylation and the extent of SLC22A8 enrichment in immune infiltrating cells as prognostic biomarkers in patients with renal clear cell carcinoma. In this study, the bioinformatics was used to analyze the expression and epigenetic alterations of SLC22A8 in RCC and its interconnection with immune cells to determine its expression pattern, potential function, and prognostic value in ccRCC, providing new insights into the clinical diagnosis, prognosis, and treatment strategies for patients with ccRCC.

## 2. Methods

### 2.1. Patient Data Sets

We obtained RNA-Seq data and clinical data in kidney renal clear cell carcinoma (KIRC) project level 3 HTSeq-FPKM format from The Cancer Genome Atlas (TCGA) database (https://portal.gdc.cancer.gov/) and then converted fragments per kilobase per million format RNA-Seq data to TPM (transcripts per million reads) format and log2 transformed. The RNA-Seq data and clinical information from 611 KIRC projects, including 72 paired neighboring tissues have been collected. In addition, the RNA-seq data in TPM format from UCSC XENA (https://xenabrowser.net/datapages/)^[21]^ processed uniformly by the Toil process for TCGA and GTEx were acquired. The KIRC of TCGA and the corresponding normal tissue data in GTEx and compared the RNA-seq data in TPM format and log2-transformed for expression between samples have been extracted successfully. Data for GSE53757 and GSE66271 were downloaded from the GEO database (https://www.ncbi.nlm.nih.gov/geo/) using the R package GEOquery package,^[22]^ and the limma package was used to perform differential analysis between the normal and tumor groups, with screening criteria of |logFC|>2 and adjusted *P* value of <.05. The expression of SLC22A8 in KIRC was extracted for comparison between such 2 groups.

### 2.2. Survival analysis

To investigate whether SLC22A8 expression levels influence the clinical outcome of ccRCC patients, a prognostic classifier to compare survival differences with a Kaplan–Meier survival curve has been constructed. Depending on the median SLC22A8 expression, patients in the test and validation groups were divided into a high SLC22A8 expression group and a low SLC22A8 expression group. The relationship between SLC22A8 expression and overall survival (OS), disease-specific survival (DSS), and progression-free interval (PFI) of patients with ccRCC was analyzed using Kaplan–Meier curves.^[23]^ Additionally, associations between SLC22A8 expression and disease-free survival (DFS) in ccRCC patients were analyzed using the GEIPA database (http://gepia.cancer-pku.cn/).^[24]^ Hazard ratios (HRs) and 95% confidence intervals (CIs) were calculated using univariate survival analysis.

### 2.3. Univariate and multivariate logistic regression analyses

To further determine the influence of SLC22A8 expression in ccRCC patients, the univariate Cox regression analysis has been used to calculate the association between the expression levels of SLC22A8 and patient OS in the 2 cohorts. Multivariate analysis was used to assess whether SLC22A8 is an independent prognostic factor for survival in patients with ccRCC. When the *P* value was <.05, SLC22A8 was considered statistically significant in the Cox regression analysis.

### 2.4. Multiomics analysis

The UALCAN database (http://ualcan.path.uab.edu/analysis-prot.html)^[25]^ is an interactive portal for the insightful analysis of TCGA gene expression data, which can be used to analyze protein expression and phosphoprotein levels in the CPTAC dataset.^[26]^ In addition, this database has been used to analyze the relationship between SLC22A8 DNA methylation levels and the clinicopathological characteristics of ccRCC patients. The DNMIVD database (http://www.unimd.org/dnmivd/)^[27]^ is an interactive DNA methylation visualization database for the methylation analysis of 23 tumors, which was used to analyze the impact of SLC22A8 DNA methylation expression levels on the prognosis of ccRCC patients. Finally, we combined the TCGA database and Illumina Human Methylation 450 methylation platform (Illumina, San Diego, CA) to analyze the correlation between molecules and the degree of methylation of DNA methylation sites, which was validated using the MethSurv database (https://biit.cs.ut.ee/methsurv/).^[28]^ Statistical significance was set at *P* < .05.

### 2.5. Comprehensive analysis of tumor microenvironment and immune cell infiltration

To elucidate the relationship between SLC22A8 expression and immune infiltration, 22 immune cell types in ccRCC were evaluated using the CIBERSORT algorithm.^[29]^ Only those samples with a CIBERSORT output of *P* < .05 were considered worthy of further analysis, and immune cell proportion histograms and immune cell correlation heat maps were drawn based on the output. Differential analysis of immune cell infiltration was performed using the “Bioconductor Limma” R package. Next, the Wilcox test was used to analyze the differences between the high and low expression groups of 47 common immune checkpoint genes in SLC22A8. The differences were considered significant at *P* < .05, and significant differences were plotted in a box plot. The tumor immune cell infiltration and immune correlation function scores were then calculated for each ccRCC sample in TCGA database using the ssGSEA method,^[30]^ and the differences in immune scores were analyzed using the “Bioconductor Limma” R package. To explore the relevance of tumor-infiltrating immune cells to survival prediction, we performed Kaplan–Meier survival analysis of SLC22A8 gene expression in ccRCC.^[31]^ HRs and 95% CIs were calculated using univariate survival analysis. As a final step, the TME components of each ccRCC sample were evaluated using ESTIMATE,^[32]^ including the immune score (immune cell infiltration), stromal score (stromal content), ESTIMATE score (combined stromal-immune score), and tumor purity. The correlation between SLC22A8 and the cancer-immune cycle and the predicted immunotherapeutic response profile was analyzed finally.

### 2.6. Correlation analysis of SLC22A8 and markers of immune cells

The relationship between SLC22A8 expression in immune cells and multiple markers was investigated using GEPIA database. The x-axis was plotted with SLC22A8 expression level and the y-axis with other related genes. In addition, we validated genes significantly associated with SLC22A8 expression in the GEPIA database using the TIMER database (http://cistrome.org/TIMER/).^[33]^

### 2.7. Correlation analysis of SLC22A8 expression and drug sensitivity

The CellMiner database (http://discover.nci.nih.gov/cellminer/)^[34,35]^ was used to perform a correlation analysis of CWH43 expression with drug sensitivity. Data processing and graphing were performed via the “impute,” “limma,” and “ggpubr” packages in R.

### 2.8. Gene enrichment analysis

To elucidate the potential molecular mechanisms underlying the role of SLC22A8 in ccRCC, the R package “DESeq2” was used to assess differentially expressed genes (DEGs) between the high and low SLC22A8 expression groups. GO term and Kyoto Encyclopedia of Gene and Genome pathway enrichment analyses were performed for DEGs using the R package “clusterProfiler.”^[36,37]^ Gene Set Enrichment Analysis (GSEA) was performed in R version 3.6.3, involving the R package “clusterProfiler” and the selected gene sets were C5: ontology gene sets and C2: curated gene sets from the Molecular Signature Database (https://www.gsea-msigdb.org/gsea/msigdb/ index). Thresholds of p.adj < 0.05, and FDR < 0.25 were used to select significantly enriched items.

### 2.9. Statistical analysis

All statistical analyses were performed using the R software (v.3.6.3). The Wilcoxon rank-sum test, chi-square test, Spearman test, Pearson test, and logistic regression were used to analyze the relationship between SLC22A8 expression and clinicopathological characteristics. Receiver operating characteristic (ROC) curves were used to analyze the expression of SLC22A8 in GTEx, adjacent KIRC tissues, and KIRC samples. OS, DSS, progression-free survival, and DFS of TCGA patients over 10 years were analyzed using Cox regression, log-rank test, and the Kaplan–Meier method. The cutoff value for SLC22A8 expression was determined using its median value. Cox proportional risk models were used for univariate and multivariate analyses to assess the relationship between clinical and genetic characteristics and OS. Based on the Cox regression model, nomograms were created using independent prognostic factors obtained from the multivariate analysis to predict the probability of survival at 1, 3, and 5 years. Statistical significance was set at *P* < .05.

## 3. Results

### 3.1. SLC22A8 expression was lower in ccRCC tissues than in normal tissues

**Figure 1.**
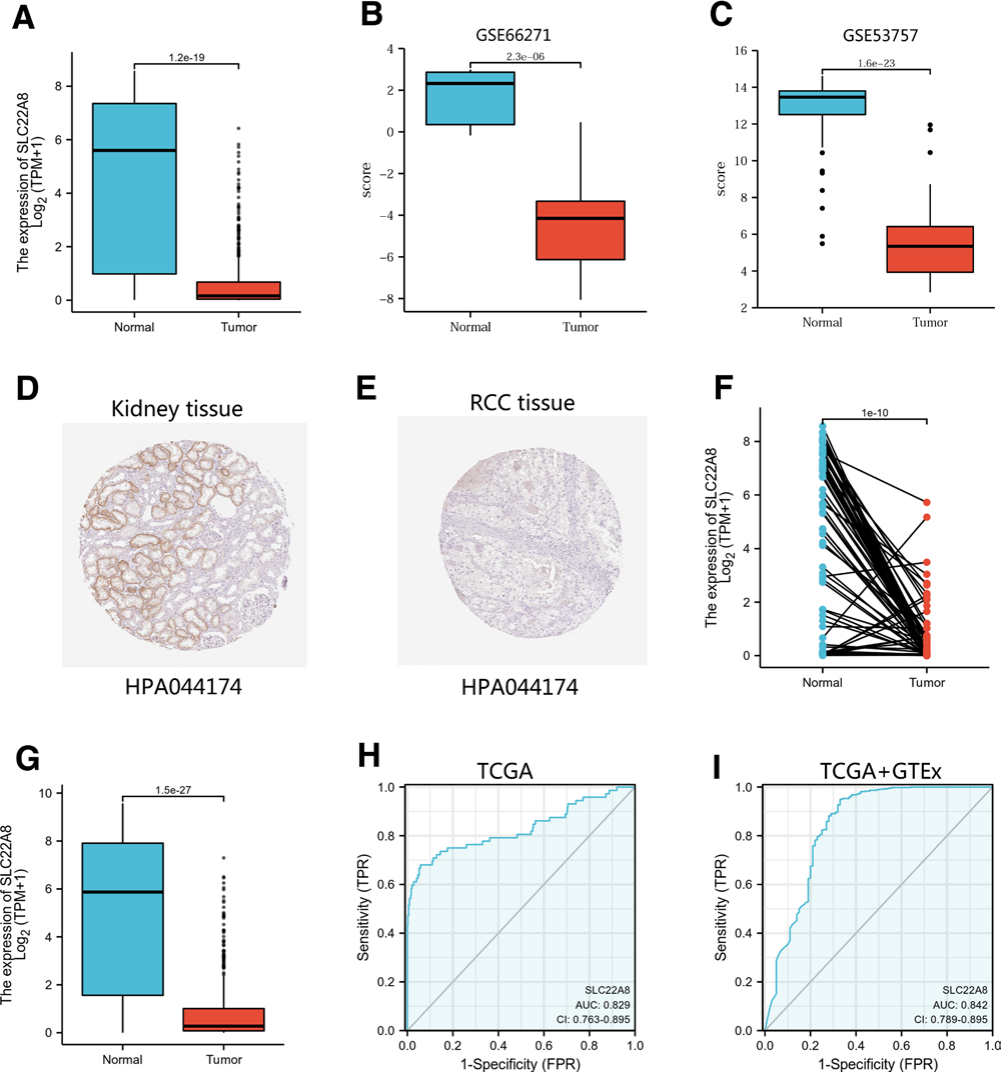
SLC22A8 expression was lower in ccRCC tissues than in normal tissues. (A) Wilcoxon rank-sum test was used to analyze the difference expression of SLC22A8 in ccRCC tissues and adjacent tissues. (B) Validation of lower SLC22A8 expression in ccRCC than that in normal tissue in GSE66271 dataset. (C) Validation of lower SLC22A8 expression in ccRCC than that in normal tissue in GSE53757 dataset. (D, E) The level of SLC22A8 protein in RCC tissue was lower than that in normal tissue in the Human Protein Atlas (antibody HPA044174, 10X). (F) Wilcoxon signed rank-sum test was used to detect the difference expression of SLC22A8 in ccRCC tissues and adjacent tissues. (G) Wilcoxon rank-sum test was used to analyze the difference expression of SLC22A8 in normal adjacent tissues of GTEx combined with TCGA and ccRCC tissues of TCGA. (H, I) ROC curve showed the efficiency of SLC22A8 expression level to distinguishing ccRCC tissue from nontumor tissue. The X-axis represents false-positive rate and Y-axis represents true positive rate. ccRCC = clear cell renal cell carcinoma, RCC = renal cell carcinoma, ROC = receiver operating characteristic.

We compared SLC22A8 expression in 72 paraneoplastic tissue samples and 539 ccRCC tissue samples from the TCGA-KIRC dataset. SLC22A8 expression was significantly lower in ccRCC tissues (*P* = 1.2e–19; Fig. 1A), which was validated in the GEO database (*P* = 2.3e–06, 1.6e–23; Fig. 1B, C). The Human Protein Atlas further confirmed that SLC22A8 protein levels were lower in kidney cancer tissues than in normal tissues (Fig. 1D, E). We also analyzed the expression of SLC22A8 in 72 ccRCC samples and matched paracancerous samples. The results showed that SLC22A8 was expressed at low levels in ccRCC tissues (*P* = 1.2e–04; Fig. 1F). The expression of SLC22A8 in normal samples of GTEx combined with adjacent ccRCC tissues and ccRCC samples was analyzed, and SLC22A8 was found to be significantly underexpressed in ccRCC (*P* = 1e−10; Fig. 1G). To determine the differential expression of SLC22A8 in renal tumors and normal tissues, the transcript levels of SLC22A8 in multiple tumor types and normal tissues were analyzed using TCGA and GTEx databases. To determine the differential expression of SLC22A8 in renal tumors and normal tissues, the transcript levels of SLC22A8 in multiple tumor types and normal tissues were analyzed using TCGA and GTEx databases. SLC22A8 expression was significantly lower in both KICH and KIRP tissues than in normal tissues (Fig. S1, Supplemental Digital Content, http://links.lww.com/MD/H119). Moreover, a ROC curve was used to analyze the effectiveness of SLC22A8 expression in paraneoplastic tissues, ccRCC samples, and normal samples of GTEx combined with adjacent ccRCC tissues and ccRCC samples. The area under the curve of SLC22A8 was >0.7, suggesting that SLC22A8 may be a potential moderate identification molecule for ccRCC tissues (Fig. 1H, I).

### 3.2. Association between SLC22A8 gene expression and clinical characteristics

**Figure 2.**
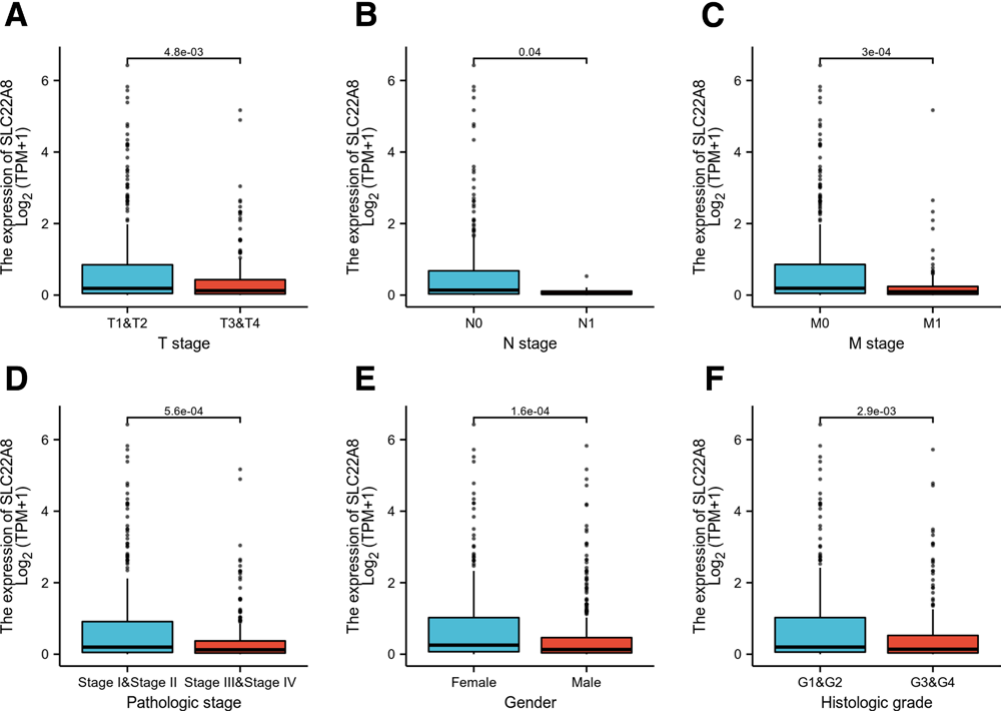
Association between SLC22A8 gene expression and clinical characteristics, including (A) T stage. (B) N stage. (C) M stage. (D) Pathologic stage. (E) Gender. (F) Histologic grade.

The clinicopathological characteristics of patients with ccRCC are shown in Table 1. We collected 539 primary ccRCC cases with clinical and gene expression data from TCGA database and divided ccRCC patients into a low expression group (n = 269) and a high expression group (n = 270) based on the mean SLC22A8 relative expression. The relationship between SLC22A8 expression levels and the clinicopathological characteristics of ccRCC was analyzed using the chi-square test, Fisher exact test, and Wilcoxon signed rank-sum test. The results showed that SLC22A8 expression was associated with T stage (*P* = 4.8e–03), N stage (*P* = .04), M stage (*P* = 3e–04), pathologic stage (*P* = 5.6e–04), sex (*P* = 1.6e–04), and histologic grade (*P* = 2.9e–03; Fig. 2), but not with other clinical features (Fig. S2, Supplemental Digital Content, http://links.lww.com/MD/H120).

**Table 1:** Correlation between SLC22A8 expression and clinicopathological characteristics in ccRCC.

Characteristic	Low expression of SLC22A8	High expression of SLC22A8	*P*
n	269	270	
Age, yr, n (%)			.518
≤60	130 (24.1%)	139 (25.8%)	
>60	139 (25.8%)	131 (24.3%)	
Gender, n (%)			<.001
Female	74 (13.7%)	112 (20.8%)	
Male	195 (36.2%)	158 (29.3%)	
Race, n (%)			.939
Asian	4 (0.8%)	4 (0.8%)	
Black or African American	30 (5.6%)	27 (5.1%)	
White	233 (43.8%)	234 (44%)	
T stage, n (%)			.004
T1	121 (22.4%)	157 (29.1%)	
T2	43 (8%)	28 (5.2%)	
T3	96 (17.8%)	83 (15.4%)	
T4	9 (1.7%)	2 (0.4%)	
N stage, n (%)			.066
N0	131 (51%)	110 (42.8%)	
N1	13 (5.1%)	3 (1.2%)	
M stage, n (%)			.006
M0	198 (39.1%)	230 (45.5%)	
M1	50 (9.9%)	28 (5.5%)	
Pathologic stage, n (%)			.003
Stage I	115 (21.5%)	157 (29.3%)	
Stage II	35 (6.5%)	24 (4.5%)	
Stage III	65 (12.1%)	58 (10.8%)	
Stage IV	51 (9.5%)	31 (5.8%)	
Primary therapy outcome, n (%)			.143
PD	6 (4.1%)	5 (3.4%)	
SD	5 (3.4%)	1 (0.7%)	
PR	0 (0%)	2 (1.4%)	
CR	58 (39.5%)	70 (47.6%)	
Histologic grade, n (%)			<.001
G1	4 (0.8%)	10 (1.9%)	
G2	107 (20.2%)	128 (24.1%)	
G3	98 (18.5%)	109 (20.5%)	
G4	54 (10.2%)	21 (4%)	
Serum calcium, n (%)			.020
Elevated	8 (2.2%)	2 (0.5%)	
Low	88 (24%)	115 (31.4%)	
Normal	82 (22.4%)	71 (19.4%)	
Hemoglobin, n (%)			.495
Elevated	1 (0.2%)	4 (0.9%)	
Low	131 (28.5%)	132 (28.8%)	
Normal	93 (20.3%)	98 (21.4%)	
Laterality, n (%)			.493
Left	130 (24.2%)	122 (22.7%)	
Right	138 (25.7%)	148 (27.5%)	
Age, median (IQR)	61 (53–70)	60 (51–69)	.235

ccRCC = clear cell renal cell carcinoma, CR = complete response, IQR = interquartile range, PD = progressive disease, PR = partial response, SD = stable disease.

Logistic regression was used to analyze the relationship between SLC22A8 expression and the clinicopathological features of ccRCC (Table 2). The results showed that SLC22A8 was significantly correlated with N stage (*P* = .048), M stage (*P* = .004), pathologic stage (*P* = .011), sex (*P* < .001), histologic grade (*P* = .032), and serum calcium (*P* = .039).

**Table 2:** SLC22A8 expression associated with clinicopathologic characteristics (logistic regression).

Characteristics	Total (N)	Odds ratio (95% CI)	*P* value
Age (>60 vs ≤60)	539	0.881 (0.628–1.236)	.464
Gender (male vs female)	539	0.535 (0.372–0.767)	<.001
Race (White vs Asian and Black or African American)	532	1.101 (0.655–1.858)	.715
T stage (T3 and T4 vs T1 and T2)	539	0.718 (0.503–1.023)	.067
N stage (N1 vs N0)	257	0.275 (0.062–0.879)	.048
M stage (M1 vs M0)	506	0.482 (0.289–0.789)	.004
Pathologic stage (stage III and stage IV vs stage I and stage II)	536	0.636 (0.447–0.902)	.011
Primary therapy outcome (CR vs PD and SD and PR)	147	1.659 (0.630–4.547)	.309
Histologic grade (G3 and G4 vs G1 and G2)	531	0 .688 (0.488–0.968)	.032
Serum calcium (low vs elevated)	213	5.227 (1.272–35.211)	.039
Hemoglobin (low vs elevated)	268	0.252 (0.013–1.731)	.220
Laterality (right vs left)	538	1.143 (0.814–1.605)	.440

CI = confidence interval, CR = complete response, PD = progressive disease, PR = partial response, SD = stable disease.

### 3.3. Low SLC22A8 gene expression was closely associated with poor prognosis of patients with ccRCC

**Figure 3.**
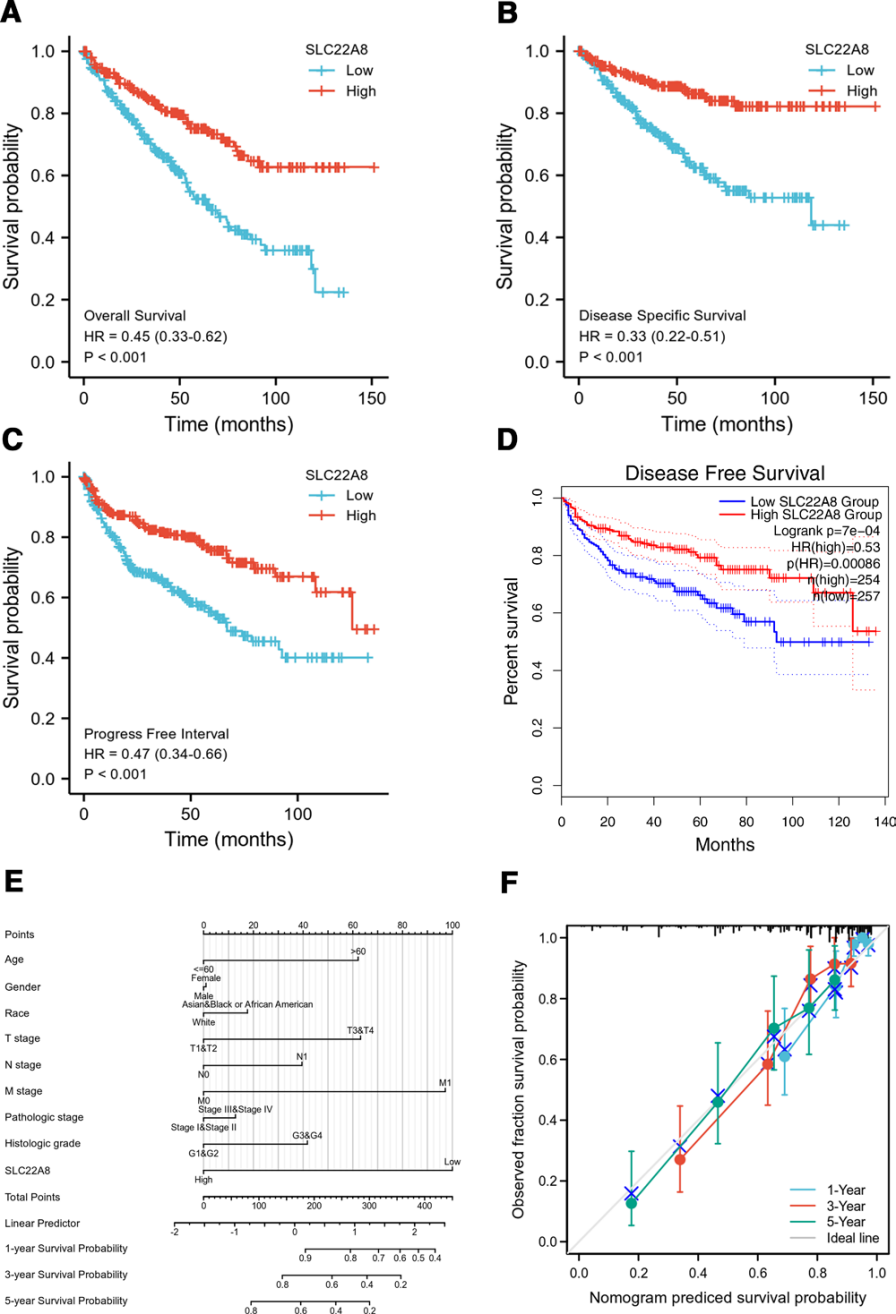
Kaplan–Meier survival analysis in comparison with the high or low level of SLC22A8 expression in ccRCC patients. (A) OS. (B) DSS. (C) PFI. (D) DFS. (E) A nomogram plot to predict 1-, 3-, and 5-yr OS probability in ccRCC patients. (F) Calibration diagram of nomogram plot that predicts ccRCC patients with 1-, 3-, and 5-yr survival probability. ccRCC = clear cell renal cell carcinoma, DFS = disease-free survival, DSS= disease-specific survival, OS = overall survival, PFI = progress free interval.

Based on SLC22A8 gene expression status, we performed survival probability via Kaplan–Meier analysis. From these results, we identified a low expression level of the SLC22A8 gene that correlates with poor OS probability (*P* < .001; Fig. 3A). Furthermore, we also performed DSS (*P* < .001; Fig. 3B), PFI (*P* < .001; Fig. 3C), and DFS (*P* = 7e–04; Fig. 3D), which was consistent with the result of OS revealing the survival benefit in ccRCC patients with higher than median expression levels of SLC22A8 gene.

In addition, we utilized the OS nomogram to predict the probability of survival of patients with ccRCC at 1, 3, and 5 years. According to the point scales at the top of the nomogram plot, we determined and summed the points with each corresponding factor to quantify the prediction of 1-, 3-, and 5-year OS probability (Fig. 3E). To verify the consistency and differentiation of internal and external data for the prediction effect of the nomogram plot, we further performed a calibration diagram that suggested good agreement between the predictions and the actual outcomes of the 1-, 3-, and 5-year survival probabilities (Fig. 3F).

Univariate and multivariate analyses were performed to explore the clinical significance of several prognostic factors. In the Cox proportional hazards regression model, we found that OS was significantly affected by some factors in both the univariate and multivariate analyses, including age (*P* < .001 in univariate analysis, *P* = .047 in multivariate analysis), M stage (*P* < .001 in univariate analysis, *P* = .002 in multivariate analysis), and high expression level of SLC22A8 (*P* < .001 in univariate analysis, *P* = .007 in multivariate analysis), which were considered independent prognostic factors for ccRCC patients (Table 3). In addition, a Cox univariate regression model was constructed based on the DSS and PFI data of ccRCC patients, in which we further confirmed that SLC22A8 expression was an independent prognostic factor for DSS (*P* < .001) and PFI (*P* = .001) in ccRCC patients (Tables S1 and S2, Supplemental Digital Content, http://links.lww.com/MD/H121).

**Table 3:** The univariate and multivariable survival analysis of SLC22A8 expression in ccRCC patients.

Characteristics	Total (N)	Univariate analysis		Multivariate analysis	
		Hazard ratio (95% CI)	*P* value	Hazard ratio (95% CI)	*P* value
Age	539				
≤60	269	Reference			
>60	270	1.765 (1.298–2.398)	<.001	2.164 (1.009–4.643)	.047
T stage	539				
T1 and T2	349	Reference			
T3 and T4	190	3.228 (2.382–4.374)	<.001	1.736 (0.710–4.249)	.227
N stage	257				
N0	241	Reference			
N1	16	3.453 (1.832–6.508)	<.001	0.280 (0.037–2.101)	.215
M stage	506				
M0	428	Reference			
M1	78	4.389 (3.212–5.999)	<.001	5.691 (1.931–16.773)	.002
Pathologic stage	536				
Stage I and stage II	331	Reference			
Stage III and stage IV	205	3.946 (2.872–5.423)	<.001		
Histologic grade	531				
G1 and G2	249	Reference			
G3 and G4	282	2.702 (1.918–3.807)	<.001	0.965 (0.415–2.243)	.934
Gender	539				
Female	186	Reference			
Male	353	0.930 (0.682–1.268)	.648		
Race	532				
Asian	8	Reference			
Black or African American and White	524	1.812 (0.253–12.963)	.554		
Serum calcium	213				
Elevated	10	Reference			
Low	203	0.197 (0.097–0.398)	<.001	1.540 (0.266–8.899)	.630
Hemoglobin	268				
Elevated	5	Reference			
Low	263	0.370 (0.117–1.171)	.091		
Laterality	538				
Left	252	Reference			
Right	286	0.706 (0.523–0.952)	.023	1.053 (0.497–2.229)	.893
SLC22A8	539				
Low	270	Reference			
High	269	0.454 (0.332–0.621)	<.001	0.320 (0.140–0.731)	.007

ccRCC = clear cell renal cell carcinoma, CI = confidence interval.

### 3.4. Multiomics analysis of SLC22A8 in ccRCC

**Figure 4.**
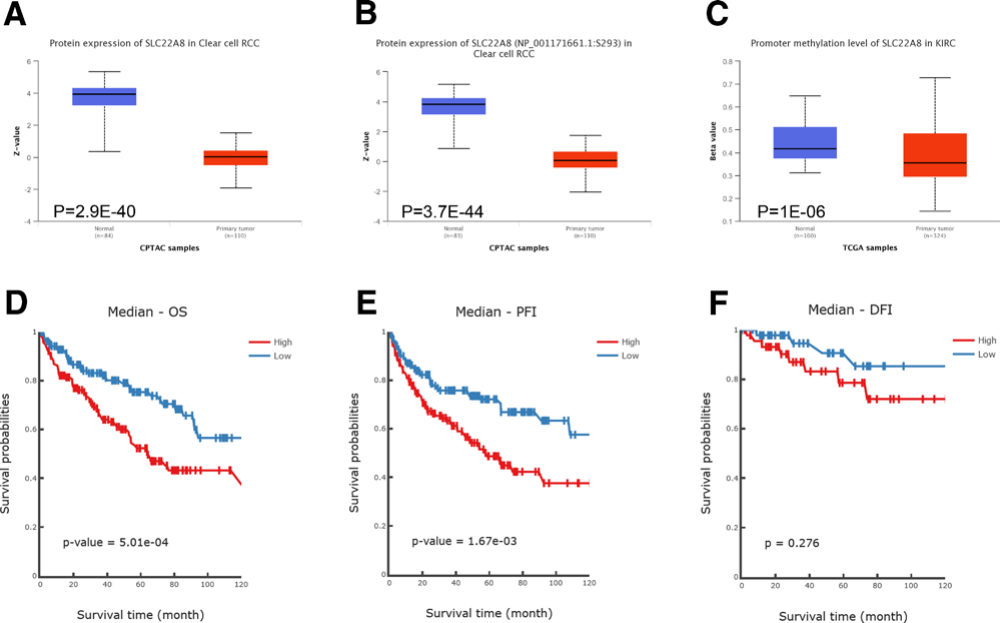
Multiomics analysis of SLC22A8 in ccRCC. (A) Analysis of SLC22A8 protein expression levels in ccRCC tissues and normal tissues using CPTAC database. (B) Analysis of protein phosphorylation expression levels of SLC22A8 in ccRCC tissues and normal tissues using CPTAC database. (C) Analysis of methylation expression levels of SLC22A8 DNA in ccRCC tissues and normal tissues using TCGA database. (D–F) Analysis of the relationship between SLC22A8 DNA methylation levels and prognosis of ccRCC patients using DNMIVD database. ccRCC = clear cell renal cell carcinoma, TCGA = The Cancer Genome Atlas.

**Figure 5.**
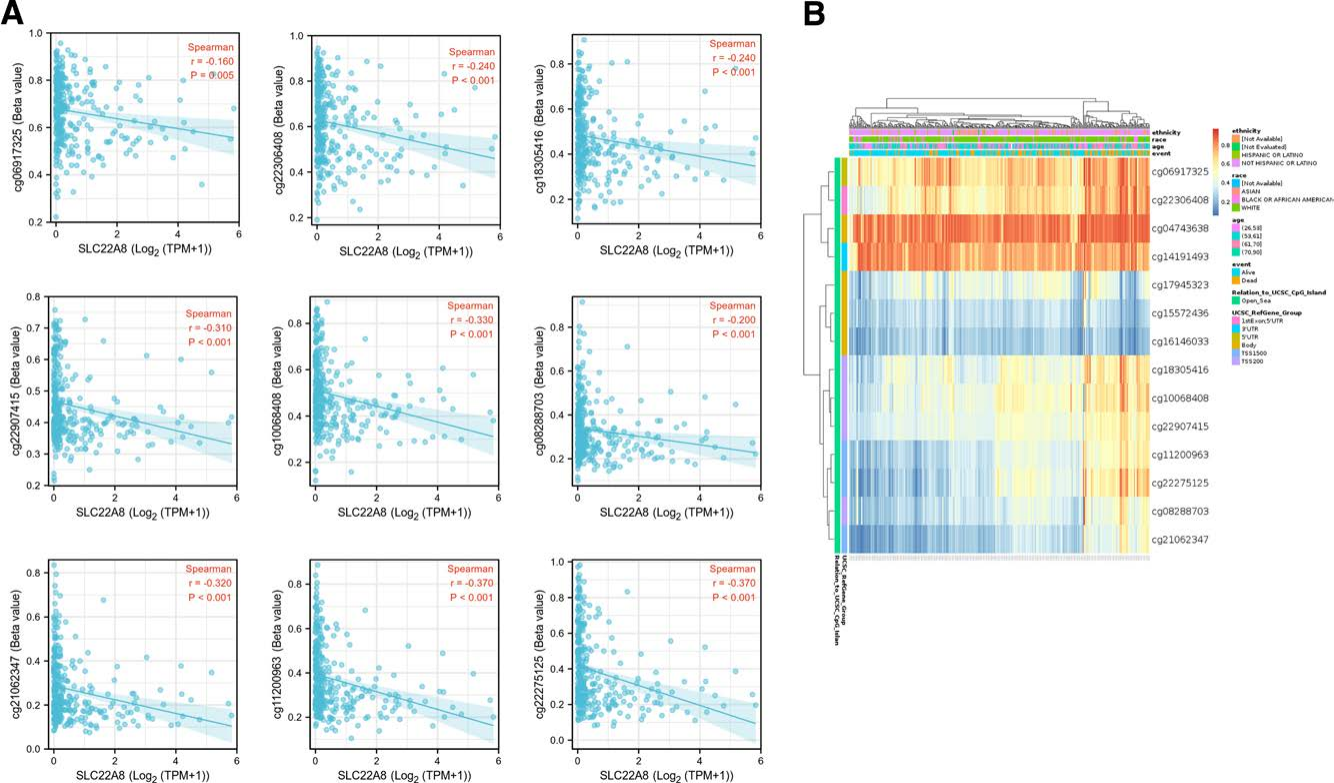
SLC22A8 DNA methylation expression levels and corresponding probe correlations. (A) The probes include cg06917325, cg22306408, cg18305416, cg22907415, cg10068408, cg08288703, cg21062347, cg11200963, and cg22275125. (B) The visualization between the methylation level and the SLC22A8 expression. (software: MethSurv, version: MethSurv©2017, URL: https://biit.cs.ut.ee/ meths urv/).

We performed multiomics analysis of SLC22A8. The CPTAC database results showed that total SLC22A8 protein was expressed at significantly lower levels in renal clear cell carcinoma tissues than in normal tissues (*P* = 2.9e–40; Fig. 4A). We also compared differences in the phosphorylation levels of SLC22A8 in normal and ccRCC tissues. The S293 site within the SLC22A8 Sugar_trN structural domain was significantly less phosphorylated in ccRCC tissues than in normal tissues (*P* = 3.7e–44; Fig. 4B). Next, we investigated the expression levels of methylation of SLC22A8 in renal clear cell carcinomas with different clinical features using the UALCAN database. The methylation level of SLC22A8 was significantly lower in ccRCC tissues than that in normal tissues (*P* = 1e–06; Fig. 4C). Moreover, we found that SLC22A8 expression was significantly different in different cancer stages, race, gender, age, tumor grade, and nodal metastasis status (Fig. S3, Supplemental Digital Content, http://links.lww.com/MD/H122). In addition, we used the MethSurv tool to investigate the prognostic value of SLC22A8 promoter methylation levels in relation to each CpG in ccRCC patients (*P* < .05; Fig. 5A). Figure 5B showed 14 methylated CpG sites.

Finally, we investigated the relationship between SLC22A8 DNA methylation levels and patient prognosis using the DNMIVD database. The median DNA methylation beta values were used as the threshold to divide the samples into high and low groups. *P* values were calculated using the log-rank test. The results showed that the 10-year OS and PFI rates were significantly lower in patients with high expression of SLC22A8 DNA methylation than in patients with low expression of SLC22A8 DNA methylation (*P* = 5.01e–04, *P* = 1.67e–03; Fig. 4D, E). Although the level of SLC22A8 DNA methylation was not significantly associated with patient disease-free interval (DFI) prognosis, it showed the same trend (*P* = .276; Fig. 4F).

### 3.5. The correlation between SLC22A8 expression and immune infiltration

**Figure 6.**
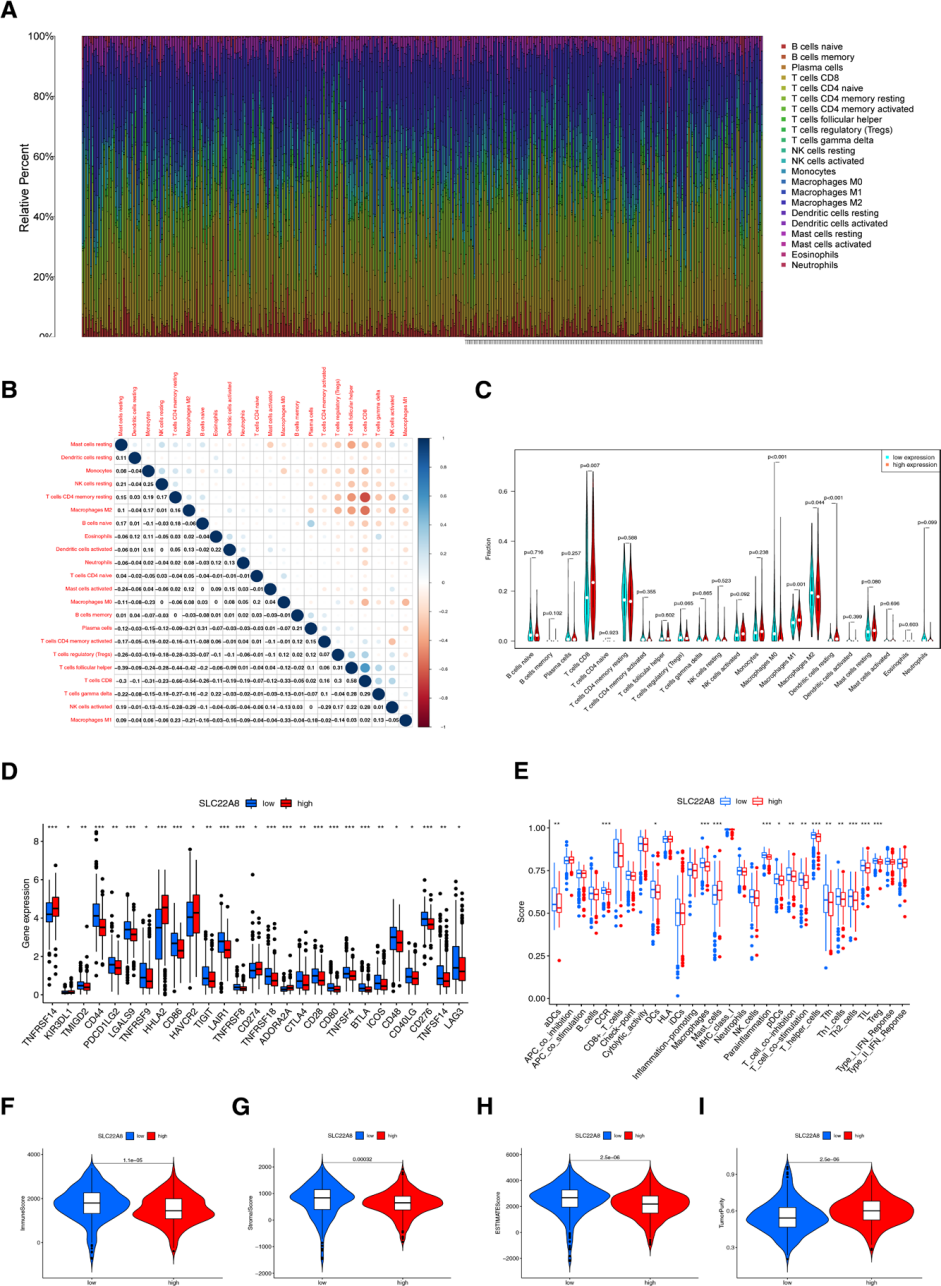
Correlation analysis of the immune infiltration pattern. (A) Bar graph showing the relative content distribution of 22 TICs in 412 ccRCC patients. Rows represent ccRCC cases. (B) Heat map showing the correlation between the 22 TICs. Differences between the high and low SLC22A8 expressing groups in (C) infiltrating immune cells, (D) differences of immune checkpoints, (E) immune function and immune cells. (F) immune scores, (G) stromal scores, (H) ESTIMATE scores, and (I) tumor purity. * *P* < .05, ** *P* < .01, *** *P* < .001. ccRCC = clear cell renal cell carcinoma, TIC = tumor-infiltrating immune cell.

To understand the relative content distribution and correlation of 22 TICs (tumor-infiltrating immune cells) in the TCGA-ccRCC cohort, we obtained the percentage of immune cell infiltration in each sample using the CIBERSORT algorithm; a total of 412 samples met the screening criteria (*P* < .05) and plotted the immune cell percentage histogram (Fig. 6A) and immune cell percentage correlation heat map for 412 samples. In ccRCC, CD8+ T cells showed a strong positive correlation with Tfh cells (*r* = 0.58, *P* < .05) and a negative correlation with CD4 memory resting cells (*r* = –0.66, *P* < .05) and M2 macrophages (*r* = –0.54, *P* < .05). Moreover, Tfh levels were negatively correlated with resting mast cells (*r* = –0.39, *P* < .05), resting CD4 memory T cells (*r* = –0.44, *P* < .05), and M2 macrophages (*r* = –0.42, *P* < .05; Fig. 6B). We also compared the proportion of immune cells between the high and low SLC22A8 expression groups in TCGA dataset. In TCGA, the SLC22A8 high expression group had higher levels of T cells CD8 (*P* = .007), macrophages M1 (*P* = .001), and resting dendritic cells (*P* < .001), while the SLC22A8 low expression group had higher levels of macrophages M0 (*P* < .001; Fig. 6C). We further analyzed the correlation between SLC22A8 expression and immune cell markers using the TIMER and GEPIA databases, including B cells, CD8+ T cells, M1/M2 macrophages, tumor-associated macrophages (TAMs), neutrophils, natural killer cells, and dendritic cells in KIRC. In addition, there are different kinds of functional T cells, such as Th1, Th2, Th9, Th17, Th22, Tfh, exhausted T cells, and regulatory T cells (Treg cells). From the results, we found that the expression level of SLC22A8 had a significant relationship with the infiltration levels of various immune cell markers, including Tfh, Th9, Treg, exhausted T cells, TAMs, and dendritic cells (Table 4).

**Table 4:** Correlation analysis between SLC22A8 and markers of immune cells in TIMER and GEPIA.

Cell type	Gene marker	None		Purity		Tumor		Normal	
		Cor	*P*	Cor	*P*	R	*P*	R	*P*
B cell	CD19	–0.108	*	–0.077	.097	–0.021	.63	0.013	.91
	CD20 (KRT20)	–0.106	*	–0.097	*	–0.004	.93	–0.0078	.95
	CD38	–0.064	.142	–0.044	.348	–0.066	.13	–0.096	.42
CD8 + T cell	CD8A	–0.054	.212	–0.037	.430	–0.059	.17	0.0082	.95
	CD8B	–0.028	.518	–0.011	.809	–0.054	.22	0.2	.088
Tfh	BCL6	–0.085	*	–0.12	*	–0.051	.25	–0.57	***
	ICOS	–0.099	*	–0.093	*	–0.075	.088	0.064	.6
	CXCR5	–0.136	**	–0.119	*	–0.081	.063	–0.0094	.94
Th1	T-bet (TBX21)	0.043	.317	0.04	.389	–0.096	*	–0.083	.49
	STAT4	–0.085	*	–0.081	.084	–0.09	*	–0.072	.55
	IL12RB2	–0.006	.899	–0.015	.748	–0.021	.63	0.31	**
	WSX1(IL27RA)	–0.112	**	–0.102	*	–0.1	*	–0.63	***
	STAT1	–0.04	.354	–0.026	.580	–0.039	.37	–0.5	***
	IFN-γ (IFNG)	–0.102	*	–0.103	*	–0.065	.14	–0.12	.32
	TNF-α (TNF)	–0.017	.698	–0.007	.884	–0.016	.72	–0.21	.076
Th2	GATA3	–0.07	.104	–0.024	.611	0.019	.67	–0.5	***
	CCR3	–0.058	.185	–0.015	.750	0.12	**	0.18	.14
	STAT6	0.074	.086	0.06	.200	–0.016	.71	–0.46	***
	STAT5A	–0.092	*	–0.067	.151	–0.021	.63	–0.07	.56
Th9	TGFBR2	0.217	***	0.217	***	0.046	.3	0.061	.61
	IRF4	–0.143	**	–0.118	*	–0.069	.11	–0.031	.8
	PU.1 (SPI1)	–0.202	***	–0.183	***	–0.11	**	–0.17	.15
Th17	STAT3	0.031	.481	0.053	.260	0.049	.26	–0.57	***
	IL-21R	–0.193	***	–0.162	***	–0.085	.051	0.067	.57
	IL-23R	–0.074	.088	–0.072	.121	–0.033	.45	–0.018	.88
	IL-17A	0.039	.367	0.064	.172	0.0044	.92	0.088	.46
Th22	CCR10	–0.035	.420	–0.009	.842	–0.054	.22	–0.29	*
	AHR	0.05	.254	0.05	.288	–0.0095	.83	–0.24	*
Treg	FOXP3	–0.212	***	–0.211	***	–0.14	**	–0.31	**
	CD25 (IL2RA)	–0.214	***	–0.219	***	–0.062	.16	0.15	.22
	CCR8	–0.163	***	–0.161	***	–0.1	*	0.13	.29
T-cell exhaustion	PD-1 (PDCD1)	–0.08	.066	–0.07	.134	–0.057	.19	0.057	.63
	CTLA4	–0.14	**	–0.141	**	–0.1	*	–0.047	.69
	LAG3	–0.099	*	–0.077	.099	–0.061	.17	–0.46	***
	TIM-3 (HAVCR2)	0.188	***	0.205	***	0.11	*	0.44	***
Macrophage	CD68	–0.082	.059	–0.124	**	–0.076	.084	0.1	.39
	CD11b (ITGAM)	–0.097	*	–0.087	.061	0.06	.17	–0.074	.53
M1	INOS (NOS2)	0.123	**	0.127	**	0.028	.53	0.084	.48
	IRF5	0.043	.319	0.041	.380	0.16	***	–0.62	***
	COX2 (PTGS2)	–0.122	**	–0.091	.051	–0.018	.68	–0.31	**
M2	CD163	–0.133	**	–0.141	**	–0.08	.068	–0.15	.2
	ARG1	0.06	.166	0.044	.346	–0.011	.81	0.27	*
	MRC1	0.065	.135	0.073	.119	0.031	.49	0.096	.42
	MS4A4A	–0.154	***	–0.15	**	–0.068	.12	–0.017	.89
TAM	CCL2	0.265	***	0.294	***	0.037	.4	–0.32	**
	CD80	–0.166	***	–0.16	***	–0.085	.051	–0.33	**
	CD86	–0.151	***	–0.157	***	–0.1	*	–0.11	.35
	CCR5	–0.068	.117	–0.053	.253	–0.072	.1	–0.12	.3
Monocyte	CD14	–0.152	***	–0.138	**	–0.085	.051	–0.18	.14
	CD16 (FCGR3B)	0.022	0.609	0	.999	–0.055	.21	0.22	.063
	CD115 (CSF1R)	–0.138	**	–0.127	**	–0.061	.16	–0.088	.46
Neutrophil	CD66b (CEACAM8)	0.055	.201	0.041	.380	–0.01	.82	–0.17	.16
	CD15 (FUT4)	0.023	.594	0.043	.353	0.025	.57	–0.11	.34
	CD11b (ITGAM)	–0.097	*	–0.087	.061	0.06	.17	–0.074	.53
Natural killer cell	XCL1	–0.1	*	–0.079	.092	–0.086	*	–0.22	.068
	CD7	–0.143	***	–0.123	**	–0.026	.56	–0.14	.25
	KIR3DL1	0.136	**	0.111	*	–0.037	.4	0.032	.79
Dendritic cell	CD1C (BDCA-1)	0.109	*	0.138	**	0.076	.082	0.025	.83
	CD141 (THBD)	0.036	.412	0.042	.366	–0.0051	.91	–0.24	*
	CD11c (ITGAX)	–0.121	**	–0.11	*	–0.0083	.85	–0.056	.64

Cor = *R* value of Spearman’s correlation, None = correlation without adjustment, Purity = correlation adjusted by purity, Tfh = follicular helper T cell, Th = T helper cell, Treg = regulatory T cell, TAM = tumor-associated macrophage.

*P < .05; **P < .01; ***P < .001.

Subsequently, we analyzed the differences in 47 immune checkpoint genes between the high and low expression groups of SLC22A8. The results showed that 21 immune checkpoint genes were significantly upregulated in the low expression group compared to the high expression group, including TMIGD2, CD44, PDCD1LG2, LGALS9, TNFRSF9, CD86, TIGIT, LAIR1, TNFRSF8, TNFRSF18, CTLA4, CD28, CD80, TNFSF4, BTLA, ICOS, CD48, CD40LG, CD276, TNFSF14, LAG3. BTLA, ICOS, CD48, CD40LG, CD276, TNFSF14, and LAG3. Six immune checkpoint genes were significantly downregulated: TNFRSF14, KIR3DL1, HHLA2, HAVCR2, CD274, and ADORA2A (Fig. 6D). Our results suggested that SLC22A8 may regulate the immune pattern of ccRCC by regulating the expression of these immune checkpoint genes. Sixteen immune cell subpopulations and 13 immune-related functions were quantified using ssGSEA to elucidate the correlation between SLC22A8 expression and immune status. We identified 14 immune functions and immune cell scores that were significantly higher in the low expression group than in the high SLC22A8 expression group in CCR, macrophages, parainflammation, T helper cells, TILs, and Tregs (*P* < .05), and while only mast cells had decreased scores (Fig. 6E). From the above results, it was observed that the immune response was generally stimulated in the SLC22A8 low expression group. Finally, the TME composition of ccRCC samples was analyzed using ESTIMATE. The results showed that the SLC22A8 low expression group had a higher immune score (Fig. 6F), stromal score (Fig. 6G), ESTIMATE score (Fig. 6H), and lower tumor purity (Fig. 6I) compared to the SLC22A8 high expression group. SLC22A8 can identify the TME components of patients.

To further clarify the correlation between diverse tumor-infiltrating immune cells and survival predictions, we performed a Kaplan–Meier survival analysis of SLC22A8 gene expression in ccRCC. Samples were divided into low- and high-scoring groups for differential expression analysis. Statistically significant plots were selected and are presented. The results indicated that low SLC22A8 levels were enriched in B cells (*P* = .00025), basophils (*P* = 1.2e–06), CD4 + memory T cells (*P* = 2.1e–05), CD8 + T cells (*P* = 1.1e–05), eosinophils (*P* = .0027), macrophages (*P* = 5.9e–07), mesenchymal stem cells (*P* = .0016), regulatory T cells (*P* = .00023), type 1 T helper cells (*P* = .0013), natural killer T cells (*P* = .049), and type 2 T helper cells (*P* = .021) with worse OS in ccRCC (Fig. S4, Supplemental Digital Content, http://links.lww.com/MD/H123).

### 3.6. Analysis of the relevant role of SLC22A8 in immunotherapy

**Figure 7.**
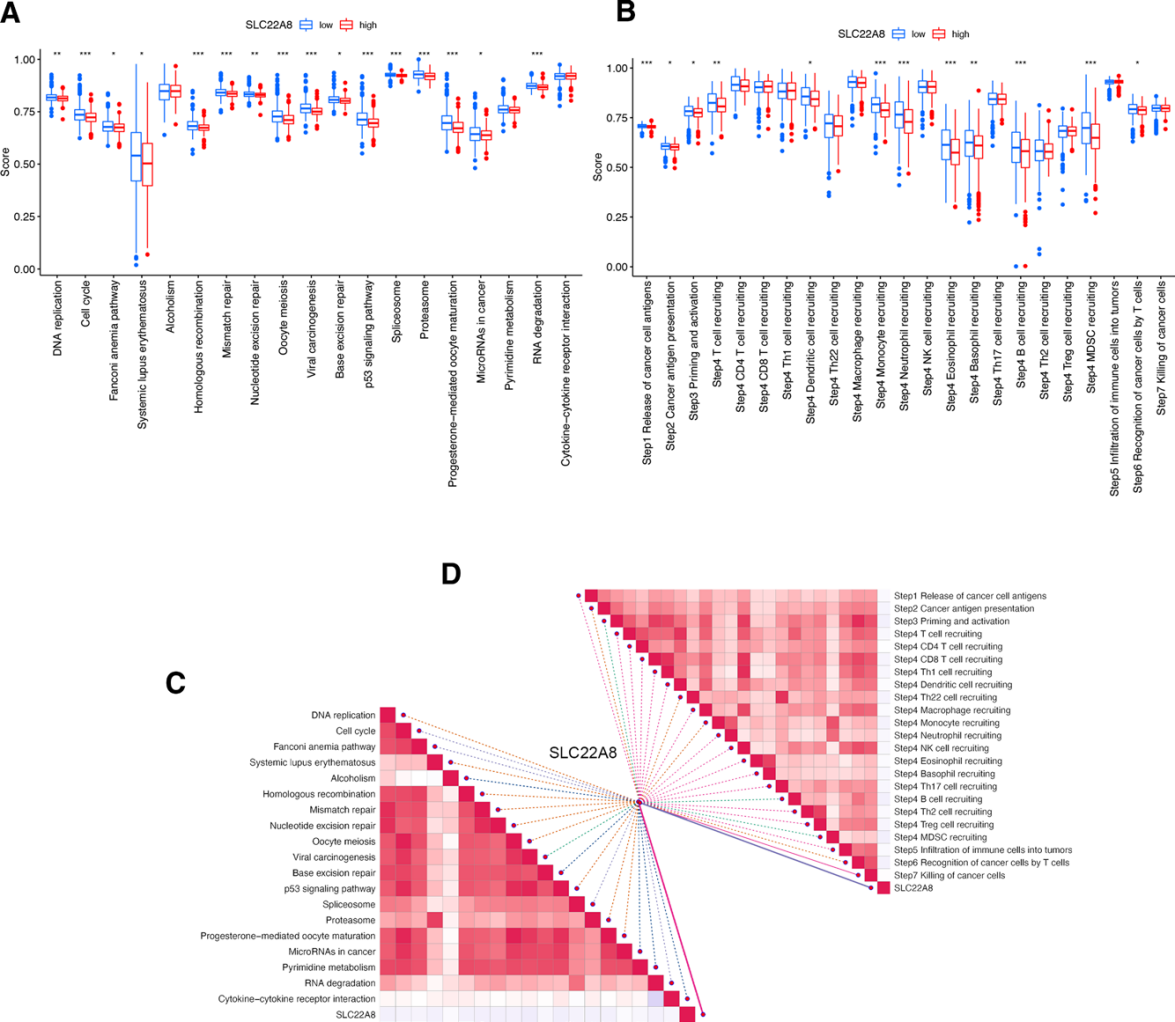
Analysis of the relevant role of SLC22A8 in immunotherapy. (A) Differences in immunotherapy clinical response scores between high and low SLC22A8 expression groups. (B) Differences in cancer-immune cycle step scores between high and low SLC22A8 expression groups. (C) Correlation of SLC22A8 with immunotherapy predicted pathway enrichment scores. (D) Correlation of SLC22A8 with cancer-immune cycle steps in ccRCC. *P < .05, **P < .01, ***P < .001. ccRCC = clear cell renal cell carcinoma.

In order to analyze the relevant role of SLC22A8 in immunotherapy, the differences in the cancer-immune cycle between high and low expression groups and observed upregulation of activity at some steps in the cycle in the low expression group was analyzed firstly, including cancer cell antigen release (step 1), cancer cell antigen expression (step 2), initiation and activation (step 3), and immune cell trafficking to the tumor (step 4) (T-cell recruitment, dendritic cell recruitment, monocyte recruitment, neutrophil granulocyte recruitment, eosinophil recruitment, basophil recruitment, B-cell recruitment, and MDSC recruitment) and recognition of cancer cells by T cells (step 6), while none of the remaining steps were significantly different (Fig. 7A). What is more, the differences in clinical response to immunotherapy between the high and low SLC22A8 expression groups were analyzed. In the low expression group, the activity of the immunotherapeutic clinical responses was upregulated, except for alcoholism, pyrimidine metabolism, and cytokine–cytokine receptor interaction, there were no significant differences were found (Fig. 7B).

In addition, the correlation between SLC22A8 expression and enrichment scores of the immunotherapy prediction pathway has been analyzed. There was no significant correlation between SLC22A8 expression and the enrichment scores of immunotherapy prediction pathways, except for alcoholism, base excision repair, microRNAs in cancer, pyrimidine metabolism, and cytokine–cytokine receptor interaction, which were all negatively correlated with the enrichment scores of immunotherapy prediction pathways (Fig. 7C). In the correlation analysis between SLC22A8 and the cancer-immune cycle, only cancer cell antigen release (step 1), cancer cell antigen expression (step 2), initiation and activation (step 3), immune cell trafficking to the tumor (step 4) (Th22 cell recruitment, monocyte recruitment, B-cell recruitment and MDSC recruitment), and recognition of cancer cells by T cells (step 6) were negatively correlated and not significantly correlated with the remaining steps (Fig. 7D).

### 3.7. Correlation between SLC22A8 gene expression levels and drug sensitivity

**Figure 8.**
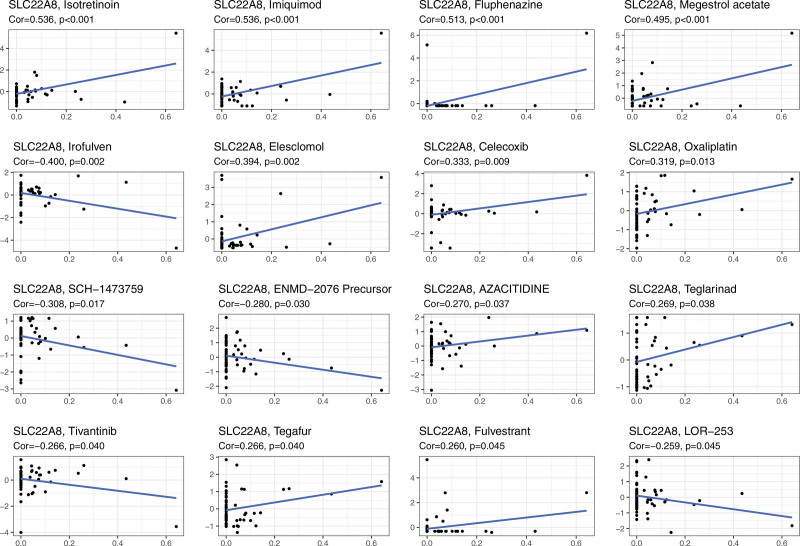
Correlation analysis of SLC22A8 expression levels with drug sensitivity. The horizontal axis indicates gene expression and the vertical axis indicates drug sensitivity.

The correlation between SLC22A8 and antitumor drug sensitivity was explored through the CellMiner database, and 16 antitumor drugs significantly correlated with CWH43 expression were screened. We found that SLC22A8 expression was significantly negatively correlated with the sensitivity of irofulven, SCH-1473759, ENMD-2076 precursor, tivantinib, and LOR-253, and with isotretinoin, imiquimod, fluphenazine, megestrol acetate, elesclomol, celecoxib, oxaliplatin, azacitidine, teglarinad, tegafur, and fulvestrant were positively correlated with sensitivity (Fig. 8).

### 3.8. GSEA identifies SLC22A8-related signaling pathways

To predict the function of SLC22A8, including related pathways, the TCGA data for DEGs between the high and low SLC22A8 expression groups have been screened. Potential functional pathways based on DEGs were further explored using clusterProfiler R package. Functional enrichment and GO analyses revealed that SLC22A8 was mainly associated with immune-related genes (Fig. 8 A, B), including active transmembrane transporter protein activity (GO:0022804), OAT protein activity (GO:0015711), metal ion transmembrane transporter protein activity (GO:0046873), monovalent inorganic cation transmembrane transporter protein activity (GO: 0015077), co-transporter protein activity (GO:0015293), and sodium ion transmembrane transporter protein activity (GO:0015081). In addition, Kyoto Encyclopedia of Gene and Genome pathway analysis revealed abundant and interfering differential genes in neuroactive ligand–receptor interactions, the PPAR signaling pathway, and parathyroid hormone synthesis, secretion, and action.

&&GSEA was used to search for GO and Reactome pathways, which revealed that the immunoglobulin complex, phagocytosis recognition, antigen binding, regulation of humoral immune response, and lymphocyte-mediated immunity were significantly enriched (Fig. 8C). In addition, CD22-mediated BCR regulation, FCGR activation, FCERI-mediated MAPK activation, and FCGR3A-mediated IL10 synthesis were significantly enriched in the reactome pathway analysis (Fig. 8D). These results suggest that SLC22A8 is associated with many malignancy-related pathways in ccRCC, particularly immune-related pathways. All the enrichment results are presented in Table S3 (Supplemental Digital Content, http://links.lww.com/MD/H124) and Table S4 (Supplemental Digital Content, http://links.lww.com/MD/H125).

## 4. Discussion

SLC22A8, which is localized on chromosome 11, encodes a protein involved in non–sodium ion transport and organic anion excretion. This protein is not only essential for the processing of metabolites, endogenous metabolites, and signaling molecules originating from intestinal microorganisms^[38]^ but also transports and removes a wide range of drugs and their metabolites. However, little is known about the expression and regulation of SLC22A8 in renal clear cell carcinoma. To the best of our knowledge, this is the first scientific study on the expression pattern of SLC22A8 in ccRCC and its predicted prognosis.

Firstly, we investigated the role of SLC22A8 expression in the tumorigenesis, progression, and prognosis of ccRCC based on various databases such as TCGA, GEO, and the Human Protein Atlas. As can be seen, SLC22A8 is lowly expressed in ccRCC compared to normal tissue (Fig. 1). Moreover, patients with low SLC22A8 expression tended to have worse OS, DSS, progression-free survival, and DFS. In addition, we constructed prognostic line graphs involving age, sex, race, TNM stage, pathological stage, and SLC22A8 expression (Fig. 2), which can be used by specialized physicians to guide and identify high-risk patients.

It has been reported that tissue-specific expression of SLC22A8 may be subject to a synergistic effect of genetic (HNF1-α and HNF1-β) and epigenetic (DNA methylation) factors.^[39]^ DNA methylation levels are known to have prognostic value in ccRCC, with a statistically significant correlation between methylation levels and clinical tumor stage, tumor differentiation, and advanced disease status.^[40]^ To further investigate the relationship between the methylation level of SLC22A8 in ccRCC and the prognosis of patients with tumors, we found that multiple sites of SLC22A8 were significantly hypomethylated in ccRCC tissues using the UALCAN database, and the degree of methylation of SLC22A8 molecules showed significant differences in tumor stage, sex, and nodal metastasis (Fig. S3, Supplemental Digital Content, http://links.lww.com/MD/H122). This suggests that SLC22A8 is epigenetically silenced in ccRCC cells. In addition, our analysis found that patients with high methylation levels of SLC22A8 had a worse prognosis. From the above study, we speculate that the degree of SLC22A8 DNA methylation may be a key factor in regulating its expression in ccRCC and that the methylation level of this molecule may be used as a prognostic indicator for patients with renal clear cell carcinoma.

The role of the TME in cancer development and progression has received increasing attention. It is well documented that ccRCC is one of the most immune-infiltrated tumors,^[20]^ however, little is known about the function and role of the SLC22A8 molecule in tumor immune relevance. In this study, we analyzed the ccRCC patient dataset from TCGA using CIBERSORT and found different levels of immune cell expression between the SLC22A8 high and low expression groups. The SLC22A8 high expression group had higher levels of T-cell CD8+, macrophage M1, and dendritic cell rest, while the SLC22A8 low expression group had higher levels of macrophage M0 levels were higher. M1-type macrophages release cytokines that inhibit the proliferation of surrounding cells, destroy adjacent tissues, and exhibit antitumor activity.^[41,42]^ In contrast, M0-type macrophage infiltration is characteristic of glioblastoma malignancy.^[43]^ Next, by examining the correlation between SLC22A8 expression and immune status, we were surprised to find that 14 immune functions and immune cell scores, including CCR, macrophages, parainflammation, T helper cells, TILs, and Tregs, were significantly higher in the low expression group than in the high SLC22A8 expression group. It is not difficult to conclude that the immune response of tumor cells was generally activated in the SLC22A8 low expression group. It can be seen that SLC22A8 can be used as a new immune-related biomarker to determine the prognosis and treatment response of ccRCC patients.

The cancer-immune cycle represents the immune response of the human immune system to cancer. The activity of the cancer-immune cycle comprehensively reflects the ultimate effect of the complex immunomodulatory interactions in TME.^[44]^ In our study, we found that most of the immune step scores appeared to be upregulated in the SLC22A8 low expression group. Also, the enrichment scores for the immunotherapy prediction pathway were generally elevated in the SLC22A8 low expression group, suggesting that patients in the SLC22A8 low expression group had a higher immune infiltration status. Our study also found a strong correlation between SLC22A8 and immune checkpoint receptor expression, with the expression of several immune checkpoints differing between the high and low SLC22A8 expression groups, such as TMIGD2, which was significantly upregulated in the low expression group. TMIGD2 is a related immune checkpoint receptor, and higher levels of TMIGD2 (a related immune checkpoint receptor) The prognosis of patients with oral squamous cell carcinoma tends to be poorer.^[45]^ Upregulation of suppressive immune checkpoint molecules, which can reduce immune cell activity, is a key feature of inflammatory TME.^[46]^ Thus, patients in the SLC22A8 low expression group were in inflammatory TME. sLC22A8 may influence the prognosis of ccRCC patients by recruiting and modulating immune cells as well as regulating the expression of immune checkpoint receptors, thus providing a theoretical basis for future combination molecular targeted immunotherapy. In conclusion, the above results strongly suggest the potential of SLC22A8 as a target for antitumor immunotherapy.

However, despite our comprehensive and systematic analysis of SLC22A8 and the use of different databases for cross- validation, limitations still exist. First, experiments are needed to validate our speculations about the potential function of SLC22A8 to increase the credibility of our results. Second, although we concluded that SLC22A8 expression is strongly associated with immune cell infiltration and patient prognosis in ccRCC, we lack direct evidence that SLC22A8 affects prognosis through its involvement in immune infiltration. In the future, prospective studies on the role of SLC22A8 in tumor immune infiltration are needed as well as the development of novel antitumor immunotherapeutic agents targeting SLC22A8.

## 5. Conclusion

In summary, this study has reported that low expression of SLC22A8 was closely associated with ccRCC progression and poor prognosis for the first time. SLC22A8 DNA methylation levels could affect the prognosis of ccRCC patients. In addition, SLC22A8 was negatively correlated with the sensitivity of several anticancer drugs, suggesting that SCL22A8 may contribute to chemoresistance. SLC22A8 was associated with immune infiltration and could influence tumor progression through abnormal tumor microenvironment and immune response. Finally, SLC22A8 would be expected to be a new prognostic biomarker for ccRCC and a potential target for immunotherapy.

The Supplementary Material (Supplemental Digital Content, http://links.lww.com/MD/H126) includes SLC22A8 expression levels in other cancers, the relationship between SLC22A8 DNA methylation levels and clinical features, and the results of enrichment analysis.

**Figure 9.**
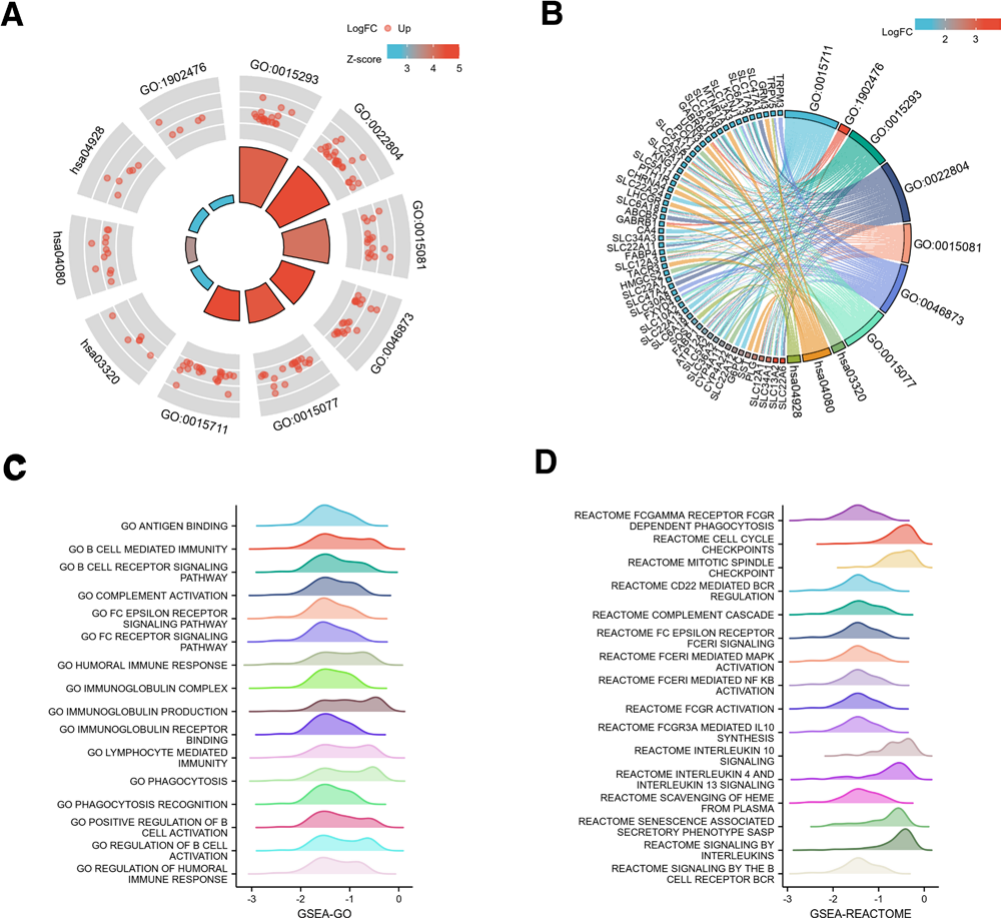
Function and pathway enrichment analyses of SLC22A8 in renal clear cell carcinoma. (A) Enrichment results for the 7 GO terms and 3 KEGG pathways. (B) Z-scores were defined as follows: upregulated genes–downregulated genes/total genes. Relationships between DEGs and the enrichment results. (C) Significant GSEA results for DEGs, including GO terms and (D) Reactome pathways. The peak height position represents the position where most of the logFC of the group of molecules is concentrated, with a negative NES and peaks to the left of zero, and a positive NES and peaks to the right of zero. GO = gene ontology, GSEA = Gene Set Enrichment Analysis, KEGG = Kyoto Encyclopedia of Gene and Genome, NES = normalize enrichment score.

## Author contributions

K.X. designed the article and wrote part of manuscript. H.C, X.Y, Y.S. and Y.W. were responsible for manuscript revision and wrote part of manuscript. Y.L. was responsible for image revision and wrote part of manuscript. X.L. and H.D. supported this manuscript and revised the manuscript. All authors have read and agreed to the published version of this manuscript.

## Supplementary Material


