# Supplementary figures and images for: SLC22A8: An indicator for tumor immune microenvironment and prognosis of ccRCC from a comprehensive analysis of bioinformatics
Source: Medicine (Baltimore). 2022 Sep 16;101(37):e30270. doi: 10.1097/MD.0000000000030270 (PMC9478252; doi:10.1097/MD.0000000000030270)

**A**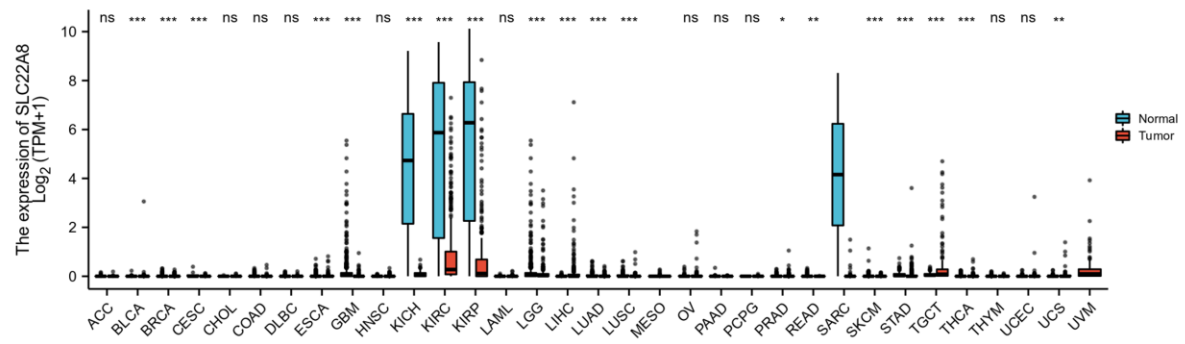**B**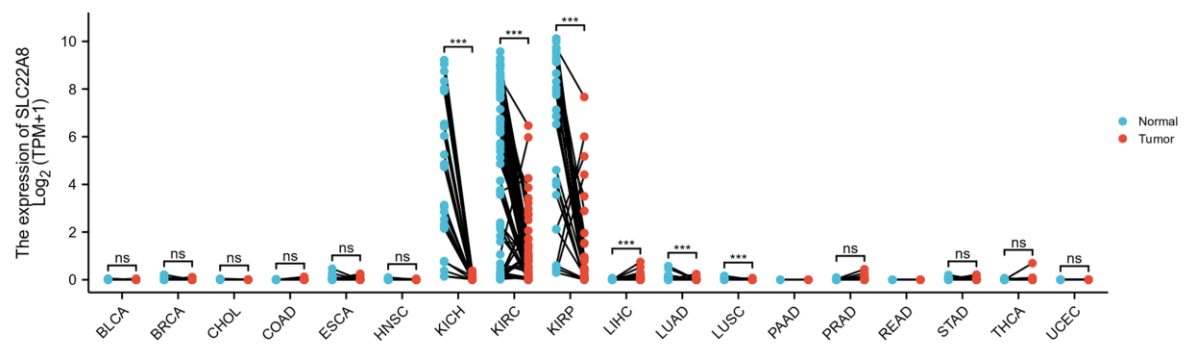

Supplement: Supplementary file 1 [file medi-101-e30270-s001.pdf]

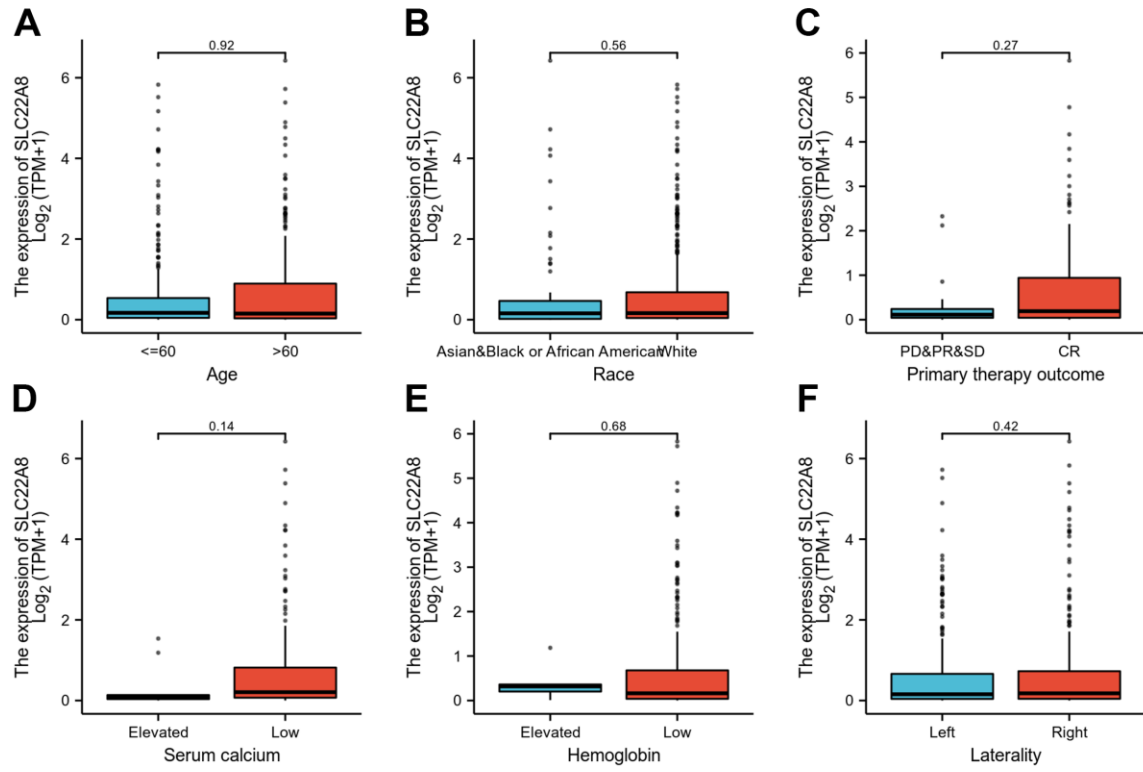

Supplement: Supplementary file 2 [file medi-101-e30270-s002.pdf]

**A**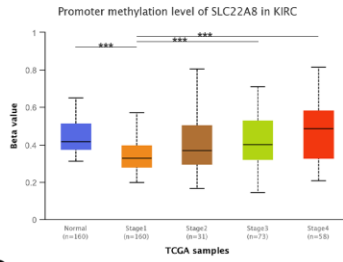**B**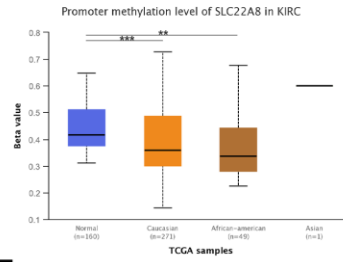**C**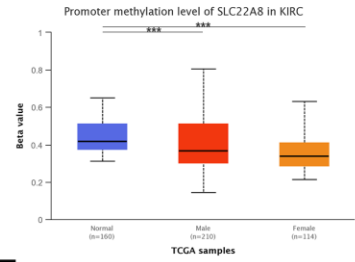**D**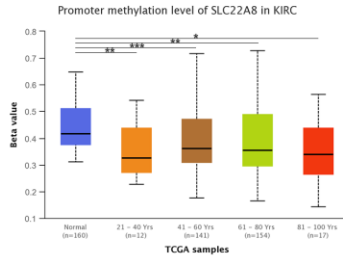**E**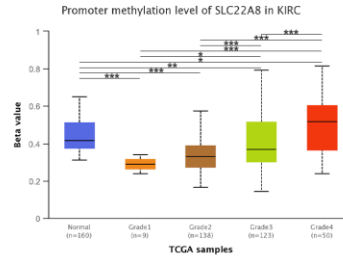**F**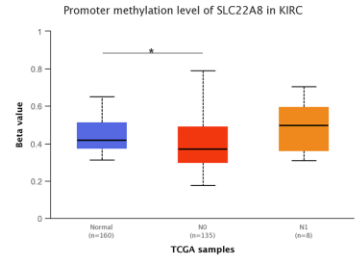

Supplement: Supplementary file 4 [file medi-101-e30270-s004.pdf]

**A**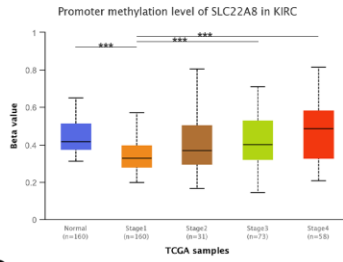**B**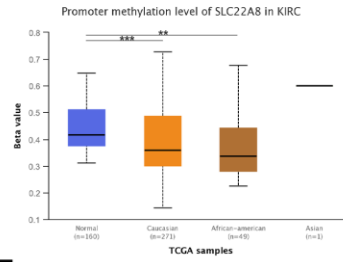**C**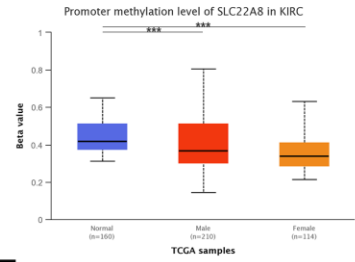**D**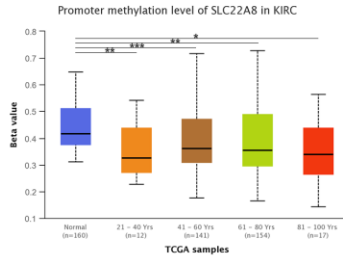**E**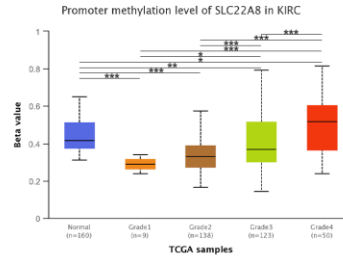**F**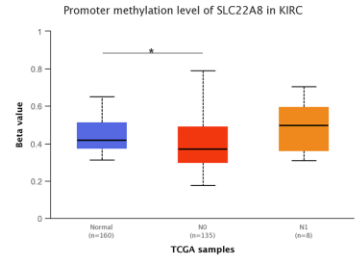

Supplement: Supplementary file 5 [file medi-101-e30270-s005.pdf]
