# Supplementary material for: SLC22A8: An indicator for tumor immune microenvironment and prognosis of ccRCC from a comprehensive analysis of bioinformatics
Source: Medicine (Baltimore). 2022 Sep 16;101(37):e30270. doi: 10.1097/MD.0000000000030270 (PMC9478252; doi:10.1097/MD.0000000000030270)
Supplement: Supplementary file 3 [file medi-101-e30270-s003.pdf]

Table 1. Correlation between SLC22A8 expression and clinicopathological characteristics in ccRCC.

Characteristic	Low expression of SLC22A8	High expression of SLC22A8	p
n	269	270	
Age, n (%)			0.518
<=60	130 (24.1%)	139 (25.8%)	
>60	139 (25.8%)	131 (24.3%)	
Gender, n (%)			< 0.001
Female	74 (13.7%)	112 (20.8%)	
Male	195 (36.2%)	158 (29.3%)	
Race, n (%)			0.939
Asian	4 (0.8%)	4 (0.8%)	
Black or African American	30 (5.6%)	27 (5.1%)	
White	233 (43.8%)	234 (44%)	
T stage, n (%)			0.004
T1	121 (22.4%)	157 (29.1%)	
T2	43 (8%)	28 (5.2%)	
T3	96 (17.8%)	83 (15.4%)	
T4	9 (1.7%)	2 (0.4%)	
N stage, n (%)			0.066
N0	131 (51%)	110 (42.8%)	
N1	13 (5.1%)	3 (1.2%)	
M stage, n (%)			0.006
M0	198 (39.1%)	230 (45.5%)	
M1	50 (9.9%)	28 (5.5%)	
Pathologic stage, n (%)			0.003
Stage I	115 (21.5%)	157 (29.3%)	
Stage II	35 (6.5%)	24 (4.5%)	
Stage III	65 (12.1%)	58 (10.8%)	
Stage IV	51 (9.5%)	31 (5.8%)	
Primary therapy outcome, n (%)			0.143
PD	6 (4.1%)	5 (3.4%)	
SD	5 (3.4%)	1 (0.7%)	
PR	0 (0%)	2 (1.4%)	
CR	58 (39.5%)	70 (47.6%)	
Histologic grade, n (%)			< 0.001
G1	4 (0.8%)	10 (1.9%)	
G2	107 (20.2%)	128 (24.1%)	
G3	98 (18.5%)	109 (20.5%)	
G4	54 (10.2%)	21 (4%)	
Serum calcium, n (%)			0.020
Elevated	8 (2.2%)	2 (0.5%)	
Low	88 (24%)	115 (31.4%)	
Normal	82 (22.4%)	71 (19.4%)	
Hemoglobin, n (%)			0.495
Elevated	1 (0.2%)	4 (0.9%)	
Low	131 (28.5%)	132 (28.8%)	
Normal	93 (20.3%)	98 (21.4%)	
Laterality, n (%)			0.493
Left	130 (24.2%)	122 (22.7%)	
Right	138 (25.7%)	148 (27.5%)	
Age, meidan (IQR)	61 (53, 70)	60 (51, 69)	0.235

Abbreviations: CR, complete response; PD, progressive disease; SD, stable disease; PR, partial response.

Table 2. SLC22A8 Expression Associated with Clinicopathologic Characteristics (Logistic Regression).

Characteristics	Total(N)	Odds Ratio(OR)	P value
Age (>60 vs. <=60)	539	0.881 (0.628-1.236)	0.464
Gender (Male vs. Female)	539	0.535 (0.372-0.767)	<0.001
Race (White vs. Asian&Black or African American)	532	1.101 (0.655-1.858)	0.715
T stage (T3&T4 vs. T1&T2)	539	0.718 (0.503-1.023)	0.067
N stage (N1 vs. N0)	257	0.275 (0.062-0.879)	0.048
M stage (M1 vs. M0)	506	0.482 (0.289-0.789)	0.004
Pathologic stage (Stage III&Stage IV vs. Stage I&Stage II)	536	0.636 (0.447-0.902)	0.011
Primary therapy outcome (CR vs. PD&SD&PR)	147	1.659 (0.630-4.547)	0.309
Histologic grade (G3&G4 vs. G1&G2)	531	0.688 (0.488-0.968)	0.032
Serum calcium (Low vs. Elevated)	213	5.227 (1.272-35.211)	0.039
Hemoglobin (Low vs. Elevated)	268	0.252 (0.013-1.731)	0.220
Laterality (Right vs. Left)	538	1.143 (0.814-1.605)	0.440

Abbreviations: CR, complete response; PD, progressive disease; SD, stable disease; PR, partial response.
