# Supplementary material for: SLC22A8: An indicator for tumor immune microenvironment and prognosis of ccRCC from a comprehensive analysis of bioinformatics
Source: Medicine (Baltimore). 2022 Sep 16;101(37):e30270. doi: 10.1097/MD.0000000000030270 (PMC9478252; doi:10.1097/MD.0000000000030270)
Supplement: Supplementary file 6 [file medi-101-e30270-s006.pdf]

Supplementary Table 3 Function and pathway enrichment analyses of SLC22A8 in renal clear cell carcinoma

Ontology	ID	Description	GeneRatio	BgRatio	pvalue	p.adjust	qvalue
BP	GO:0015711	organic anion transport	22/153	482/18670	6.62e-11	1.11e-07	9.98e-08
BP	GO:1902476	chloride transmembrane transport	5/153	88/18670	0.001	0.045	0.041
MF	GO:0022804	active transmembrane transporter activity	25/150	362/17697	4.95e-16	3.46e-14	2.49e-14
MF	GO:0015081	sodium ion transmembrane transporter activity	16/150	149/17697	1.47e-13	8.58e-12	6.17e-12
MF	GO:0046873	metal ion transmembrane transporter activity	21/150	438/17697	1.36e-10	5.96e-09	4.28e-09
MF	GO:0015077	monovalent inorganic cation transmembrane transporter activity	19/150	382/17697	6.01e-10	2.10e-08	1.51e-08
KEGG	hsa03320	PPAR signaling pathway	6/85	78/8076	1.59e-04	0.016	0.014
KEGG	hsa04080	Neuroactive ligand-receptor interaction	12/85	341/8076	2.16e-04	0.016	0.014
KEGG	hsa04928	Parathyroid hormone synthesis, secretion and action	6/85	106/8076	8.38e-04	0.020	0.019
