# Supplementary material for: SLC22A8: An indicator for tumor immune microenvironment and prognosis of ccRCC from a comprehensive analysis of bioinformatics
Source: Medicine (Baltimore). 2022 Sep 16;101(37):e30270. doi: 10.1097/MD.0000000000030270 (PMC9478252; doi:10.1097/MD.0000000000030270)
Supplement: Supplementary file 7 [file medi-101-e30270-s007.pdf]

Supplementary Table 4 GSEA analysis results

GSEA-GO

ID	ES	NES	p.adjust	FDR
REACTOME_CD22_MEDIATED_BCR_REGULATION	-0.94	-2.515	0.023	0.019
REACTOME_FCGR_ACTIVATION	-0.907	-2.443	0.0231	0.019
REACTOME_SCAVENGING_OF_HEME_FROM_PLASMA	-0.906	-2.44	0.023	0.019
REACTOME_FCERI_MEDIATED_MAPK_ACTIVATION	-0.877	-2.407	0.023	0.019
REACTOME_FCERI_MEDIATED_NF_KB_ACTIVATION	-0.844	-2.391	0.023	0.019
REACTOME_FCGR3A_MEDIATED_IL10_SYNTHESIS	-0.846	-2.34	0.023	0.019
REACTOME_SIGNALING_BY_THE_B_CELL_RECEPTOR_BCR_	-0.798	-2.294	0.023	0.019
REACTOME_COMPLEMENT_CASCADE	-0.813	-2.273	0.023	0.019
REACTOME_FCGAMMA_RECEPTOR_FCGR_DEPENDENT_PHAGOCYTOSIS	-0.78	-2.219	0.023	0.019
REACTOME_FC_EPSILON_RECEPTOR_FCERI_SIGNALING	-0.766	-2.208	0.023	0.019
REACTOME_INTERLEUKIN_10_SIGNALING	-0.619	-1.607	0.023	0.019
REACTOME_MISCELLANEOUS_TRANSPORT_AND_BINDING_EVENTS	-0.667	-1.59	0.049	0.041
REACTOME_INTERLEUKIN_4_AND_INTERLEUKIN_13_SIGNALING	-0.564	-1.572	0.023	0.019
REACTOME_SENESCENCE_ASSOCIATED_SECRETORY_PHENOTYPE_SASP	-0.551	-1.534	0.023	0.019
REACTOME_MITOTIC_SPINDLE_CHECKPOINT	-0.524	-1.46	0.034	0.029
REACTOME_CELL_CYCLE_CHECKPOINTS	-0.484	-1.418	0.034	0.029
REACTOME_SIGNALING_BY_INTERLEUKINS	-0.451	-1.338	0.023	0.019

GSEA-Reactome

ID	ES	NES	p.adjust	FDR
GO_ANTIGEN_BINDING	-0.804	-2.332	0.04	0.035
GO_B_CELL_MEDIATED_IMMUNITY	-0.782	-2.284	0.04	0.035
GO_B_CELL_RECEPTOR_SIGNALING_PATHWAY	-0.775	-2.22	0.04	0.035
GO_COMPLEMENT_ACTIVATION	-0.816	-2.37	0.04	0.035
GO_FC_EPSILON_RECEPTOR_SIGNALING_PATHWAY	-0.779	-2.263	0.04	0.035
GO_FC_RECEPTOR_SIGNALING_PATHWAY	-0.682	-1.995	0.04	0.035
GO_HUMORAL_IMMUNE_RESPONSE	-0.731	-2.158	0.04	0.035
GO_IMMUNOGLOBULIN_COMPLEX	-0.891	-2.583	0.04	0.035
GO_IMMUNOGLOBULIN_PRODUCTION	-0.733	-2.139	0.04	0.035
GO_IMMUNOGLOBULIN_RECEPTOR_BINDING	-0.886	-2.443	0.04	0.035
GO_LYMPHOCYTE_MEDIATED_IMMUNITY	-0.696	-2.053	0.04	0.035
GO_PHAGOCYTOSIS	-0.674	-1.99	0.04	0.035
GO_PHAGOCYTOSIS_RECOGNITION	-0.854	-2.378	0.04	0.035
GO_POSITIVE_REGULATION_OF_B_CELL_ACTIVATION	-0.765	-2.206	0.04	0.035
GO_REGULATION_OF_B_CELL_ACTIVATION	-0.72	-2.093	0.04	0.035
GO_REGULATION_OF_HUMORAL_IMMUNE_RESPONSE	-0.8	-2.303	0.04	0.035
