# Supplementary material for: SLC22A8: An indicator for tumor immune microenvironment and prognosis of ccRCC from a comprehensive analysis of bioinformatics
Source: Medicine (Baltimore). 2022 Sep 16;101(37):e30270. doi: 10.1097/MD.0000000000030270 (PMC9478252; doi:10.1097/MD.0000000000030270)
Supplement: Supplementary file 8 [file medi-101-e30270-s008.pdf]

Supplementary Figure

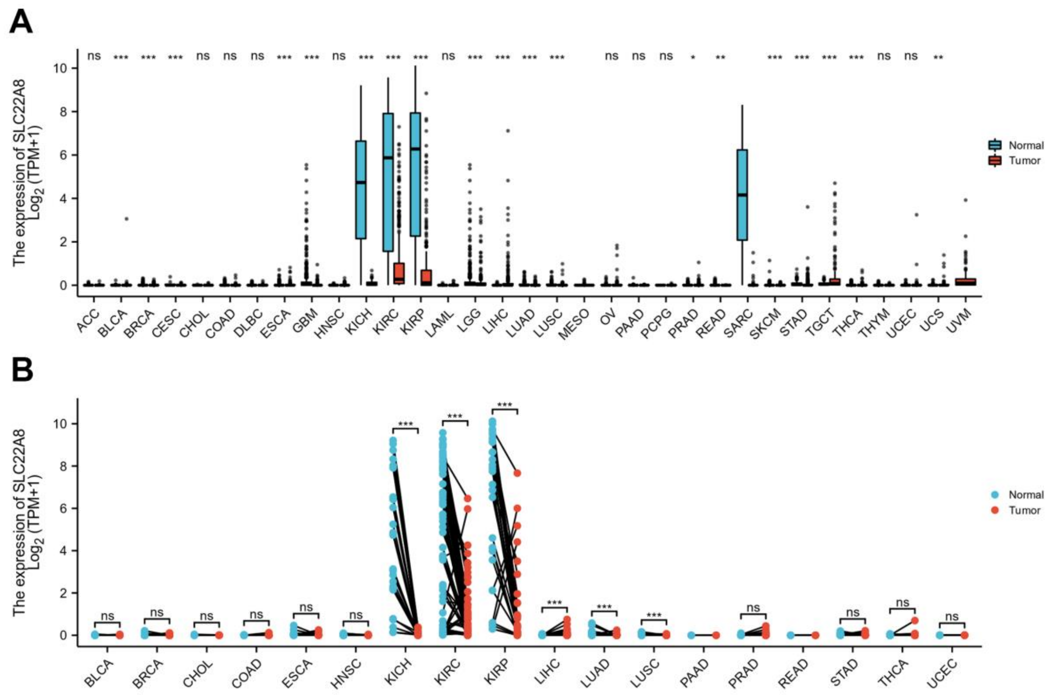

Supplementary Figure 1 (A) Wilcoxon rank sum test was used to analyze the difference expression of SLC22A8 in normal samples of GTEx combined with TCGA and tumor samples of TCGA. **(B)** Wilcoxon signed rank sum test was used to detect the difference expression of SLC22A8 in tumor tissues and their matched adjacent tissues. ns, $p \geq 0.05$; *, $p < 0.05$; **, $p < 0.01$; ***, $p < 0.001$.

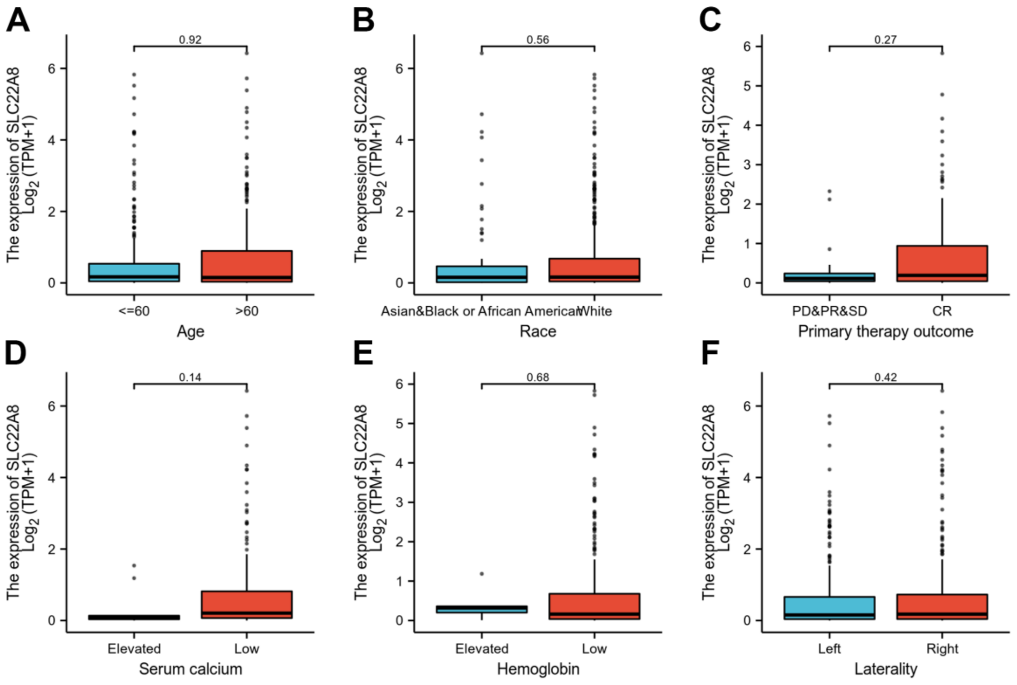

Supplementary Figure 2 Association between SLC22A8 gene expression and clinical characteristics, including (**A**) Age, (**B**) Race, (**C**) Primary therapy outcome, (**D**) serum calcium, (**E**) Hemoglobin, (**F**) Laterality. CR, complete response; PD, progressive disease; SD, stable disease; PR, partial response.

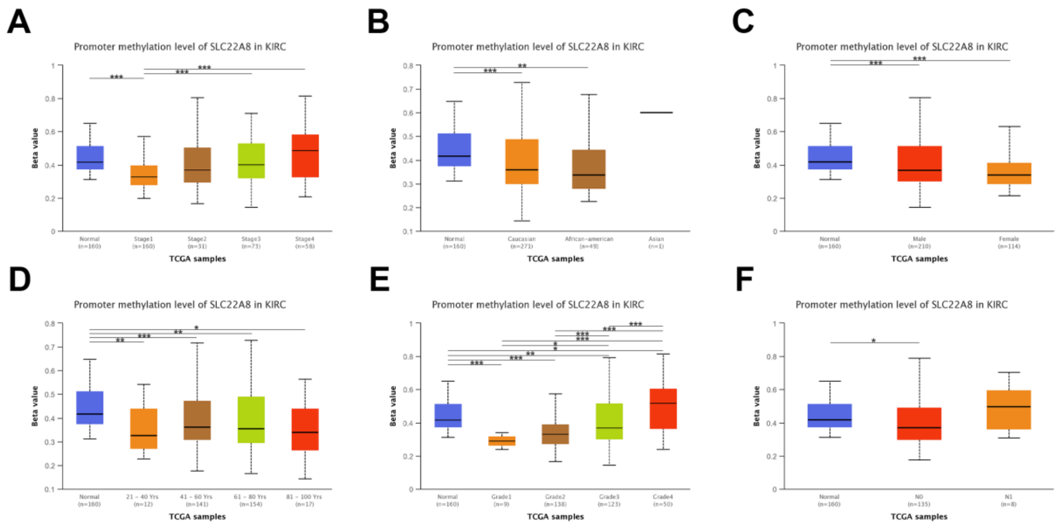

Supplementary Figure 3 Methylation levels of SLC22A8 in ccRCC patients (**A**) individual cancer stage (n=482), (**B**) race (n=481), (**C**) gender (n=484), (**D**) age (n=484), (**E**) tumor grade (n=480) and (**F**) nodal metastatic status (n=303). (*P<0.001, **P<0.01, *P<0.05).

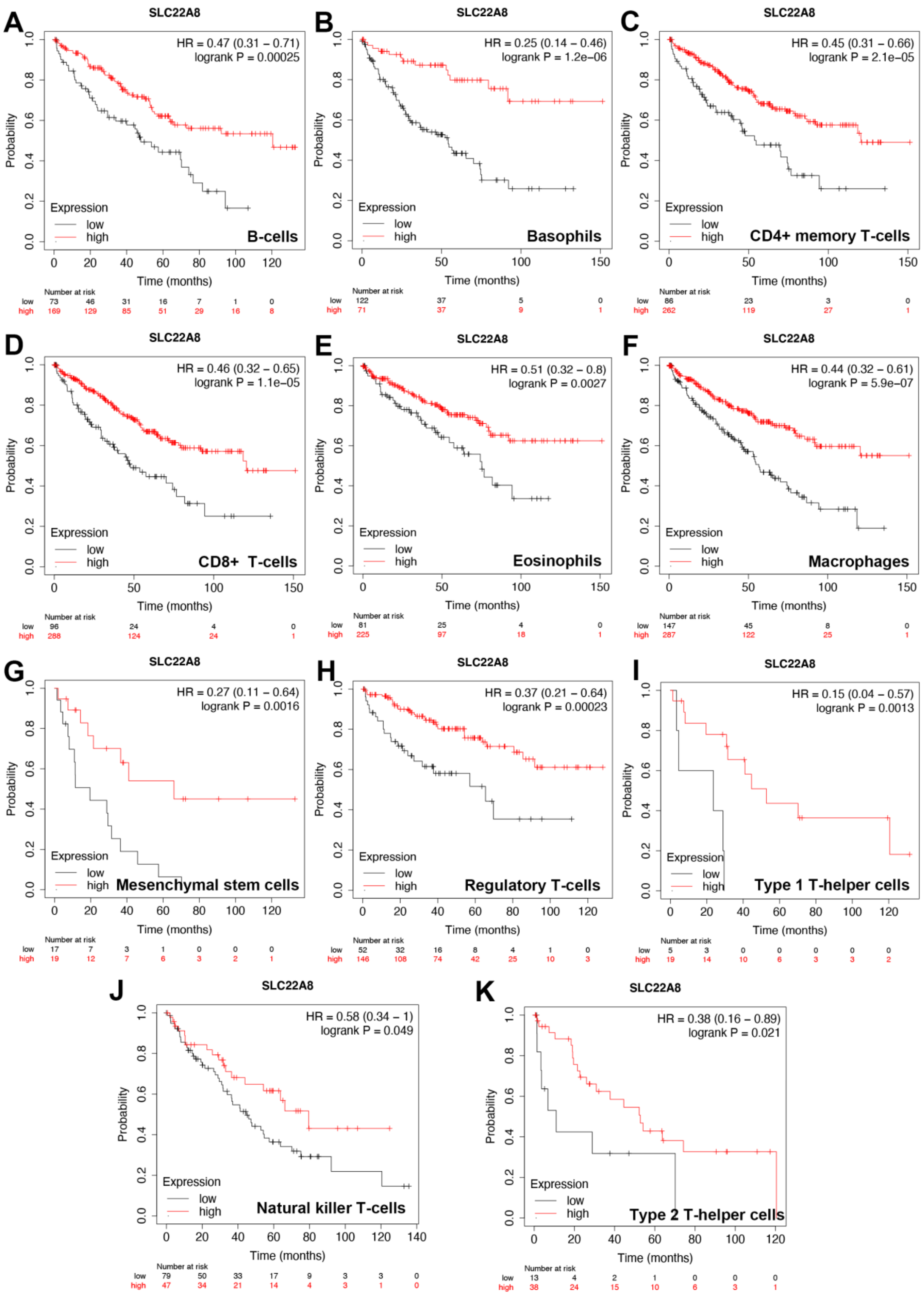

Supplementary Figure 4 Kaplan-Meier survival curves of immune cells subgroups based on high and low SLC22A8 gene expression in KIRC to assess the survival function, following B cells (**A**), Basophils (**B**), CD4+ memory T-cells (**C**), CD8+ T-cells (**D**), Eosinophils (**E**), Macrophages (**F**), Mesenchymal stem cells (**G**), Regulatory T-cells (**H**), Type 1 T-helper cells (**I**), Natural killer

T-cells (**J**), Type 2 T-helper cells (**K**).

Supplementary Table

Supplementary Table 1 The univariate and multivariable survival analysis of SLC22A8 expression in ccRCC patients.(DSS)

Characteristics	Total(N)	Univariate analysis		Multivariate analysis	
		Hazard ratio (95% CI)	P value	Hazard ratio (95% CI)	P value
Age	528				
<=60	265	Reference			
>60	263	1.335 (0.914-1.950)	0.135		
Gender	528				
Female	180	Reference			
Male	348	1.220 (0.807-1.845)	0.346		
Race	521				
Asian&Black or African American	64	Reference			
White	457	1.369 (0.635-2.951)	0.423		
T stage	528				
T1&T2	346	Reference			
T3&T4	182	5.542 (3.652-8.411)	**<0.001**	1.513 (0.644-3.551)	0.342
N stage	255				
N0	240	Reference			
N1	15	3.852 (1.825-8.132)	**<0.001**	1.205 (0.552-2.629)	0.640
M stage	495				
M0	421	Reference			
M1	74	9.108 (6.209-13.361)	**<0.001**	3.296 (1.764-6.160)	**<0.001**

Characteristics	Total(N)	Univariate analysis		Multivariate analysis	
		Hazard ratio (95% CI)	P value	Hazard ratio (95% CI)	P value
Pathologic stage	525				
Stage I&Stage II	328	Reference			
Stage III&Stage IV	197	9.835 (5.925-16.325)	**<0.001**	2.584 (0.861-7.760)	0.091
Histologic grade	520				
G1&G2	248	Reference			
G3&G4	272	4.793 (2.889-7.952)	**<0.001**	1.637 (0.805-3.332)	0.174
Laterality	527				
Left	248	Reference			
Right	279	0.637 (0.435-0.932)	**0.020**	1.050 (0.601-1.833)	0.864
SLC22A8	528				
Low	263	Reference			
High	265	0.335 (0.221-0.508)	**<0.001**	0.297 (0.151-0.585)	**<0.001**

Supplementary Table 2 The univariate and multivariable survival analysis of SLC22A8 expression in ccRCC patients.(PFI)

Characteristics	Total(N)	Univariate analysis		Multivariate analysis	
		Hazard ratio (95% CI)	P value	Hazard ratio (95% CI)	P value
Age	537				
<=60	268	Reference			
>60	269	1.275 (0.934-1.742)	0.126		
Gender	537				
Female	185	Reference			
Male	352	1.515 (1.067-2.151)	**0.020**	1.256 (0.797-1.980)	0.325
Race	530				
Asian&Black or African American	65	Reference			
White	465	1.220 (0.690-2.156)	0.495		
T stage	537				
T1&T2	349	Reference			
T3&T4	188	4.522 (3.271-6.253)	**<0.001**	1.217 (0.594-2.494)	0.592
N stage	256				
N0	240	Reference			
N1	16	3.682 (1.891-7.167)	**<0.001**	0.987 (0.482-2.020)	0.972
M stage	504				
M0	428	Reference			
M1	76	8.968 (6.464-12.442)	**<0.001**	4.109 (2.363-7.146)	**<0.001**
Pathologic stage	534				
Stage I&Stage II	331	Reference			

Characteristics	Total(N)	Univariate analysis		Multivariate analysis	
		Hazard ratio (95% CI)	P value	Hazard ratio (95% CI)	P value
Stage III&Stage IV	203	6.817 (4.770-9.744)	**<0.001**	3.158 (1.296-7.696)	**0.011**
Histologic grade	529				
G1&G2	249	Reference			
G3&G4	280	3.646 (2.503-5.310)	**<0.001**	1.536 (0.908-2.598)	0.110
Laterality	536				
Left	250	Reference			
Right	286	0.651 (0.476-0.891)	**0.007**	0.816 (0.516-1.291)	0.385
SLC22A8	537				
Low	270	Reference			
High	267	0.476 (0.344-0.658)	**<0.001**	0.434 (0.262-0.718)	**0.001**

Supplementary Table 3 Function and pathway enrichment analyses of SLC22A8 in renal clear cell carcinoma.

Ontology	ID	Description	GeneRatio	BgRatio	pvalue	p.adjust	qvalue
BP	GO:0015711	organic anion transport	22/153	482/18670	6.62e-11	1.11e-07	9.98e-08
BP	GO:1902476	chloride transmembrane transport	5/153	88/18670	0.001	0.045	0.041
MF	GO:0022804	active transmembrane transporter activity	25/150	362/17697	4.95e-16	3.46e-14	2.49e-14
MF	GO:0015081	sodium ion transmembrane transporter activity	16/150	149/17697	1.47e-13	8.58e-12	6.17e-12
MF	GO:0046873	metal ion transmembrane transporter activity	21/150	438/17697	1.36e-10	5.96e-09	4.28e-09
MF	GO:0015077	monovalent inorganic cation transmembrane transporter activity	19/150	382/17697	6.01e-10	2.10e-08	1.51e-08
KEGG	hsa03320	PPAR signaling pathway	6/85	78/8076	1.59e-04	0.016	0.014
KEGG	hsa04080	Neuroactive ligand-receptor interaction	12/85	341/8076	2.16e-04	0.016	0.014
KEGG	hsa04928	Parathyroid hormone synthesis, secretion and action	6/85	106/8076	8.38e-04	0.020	0.019

Supplementary Table 4 GSEA analysis results.

GSEA-GO

ID	ES	NES	p.adjust	FDR
REACTOME_CD22_MEDIATED_BCR_REGULATION	-0.94	-2.515	0.023	0.019
REACTOME_FCGR_ACTIVATION	-0.907	-2.443	0.0231	0.019
REACTOME_SCAVENGING_OF_HEME_FROM_PLASMA	-0.906	-2.44	0.023	0.019
REACTOME_FCERI_MEDIATED_MAPK_ACTIVATION	-0.877	-2.407	0.023	0.019
REACTOME_FCERI_MEDIATED_NF_KB_ACTIVATION	-0.844	-2.391	0.023	0.019
REACTOME_FCGR3A_MEDIATED_IL10_SYNTHESIS	-0.846	-2.34	0.023	0.019
REACTOME_SIGNALING_BY_THE_B_CELL_RECEPTOR_BCR_	-0.798	-2.294	0.023	0.019
REACTOME_COMPLEMENT_CASCADE	-0.813	-2.273	0.023	0.019
REACTOME_FCGAMMA_RECEPTOR_FCGR_DEPENDENT_PHAGOCYTOSIS	-0.78	-2.219	0.023	0.019
REACTOME_FC_EPSILON_RECEPTOR_FCERI_SIGNALING	-0.766	-2.208	0.023	0.019
REACTOME_INTERLEUKIN_10_SIGNALING	-0.619	-1.607	0.023	0.019
REACTOME_MISCELLANEOUS_TRANSPORT_AND_BINDING_EVENTS	-0.667	-1.59	0.049	0.041
REACTOME_INTERLEUKIN_4_AND_INTERLEUKIN_13_SIGNALING	-0.564	-1.572	0.023	0.019
REACTOME_SENESCENCE_ASSOCIATED_SECRETORY_PHENOTYPE_SASP	-0.551	-1.534	0.023	0.019
REACTOME_MITOTIC_SPINDLE_CHECKPOINT	-0.524	-1.46	0.034	0.029
REACTOME_CELL_CYCLE_CHECKPOINTS	-0.484	-1.418	0.034	0.029
REACTOME_SIGNALING_BY_INTERLEUKINS	-0.451	-1.338	0.023	0.019

GSEA-Reactome

ID	ES	NES	p.adjust	FDR
GO_ANTIGEN_BINDING	-0.804	-2.332	0.04	0.035
GO_B_CELL_MEDIATED_IMMUNITY	-0.782	-2.284	0.04	0.035
GO_B_CELL_RECEPTOR_SIGNALING_PATHWAY	-0.775	-2.22	0.04	0.035
GO_COMPLEMENT_ACTIVATION	-0.816	-2.37	0.04	0.035
GO_FC_EPSILON_RECEPTOR_SIGNALING_PATHWAY	-0.779	-2.263	0.04	0.035
GO_FC_RECEPTOR_SIGNALING_PATHWAY	-0.682	-1.995	0.04	0.035
GO_HUMORAL_IMMUNE_RESPONSE	-0.731	-2.158	0.04	0.035
GO_IMMUNOGLOBULIN_COMPLEX	-0.891	-2.583	0.04	0.035
GO_IMMUNOGLOBULIN_PRODUCTION	-0.733	-2.139	0.04	0.035
GO_IMMUNOGLOBULIN_RECEPTOR_BINDING	-0.886	-2.443	0.04	0.035
GO_LYMPHOCYTE_MEDIATED_IMMUNITY	-0.696	-2.053	0.04	0.035
GO_PHAGOCYTOSIS	-0.674	-1.99	0.04	0.035
GO_PHAGOCYTOSIS_RECOGNITION	-0.854	-2.378	0.04	0.035
GO_POSITIVE_REGULATION_OF_B_CELL_ACTIVATION	-0.765	-2.206	0.04	0.035
GO_REGULATION_OF_B_CELL_ACTIVATION	-0.72	-2.093	0.04	0.035
GO_REGULATION_OF_HUMORAL_IMMUNE_RESPONSE	-0.8	-2.303	0.04	0.035
